# Sludge compost: a double-edged sword for depleted soil restoration revealed by integrated multi-omics analysis

**DOI:** 10.3389/fmicb.2026.1731456

**Published:** 2026-05-22

**Authors:** Xiaojie Mei, Wenqing Wu, Ning Fang, Yali Guo, Xiaohu Dai

**Affiliations:** 1 Shanghai Investigation, Design & Research Institute Co. Ltd., Shanghai, China; 2 China Three Gorges Corporation, National Engineering Research Center of Eco-Environment in the Yangtze River Economic Belt, Wuhan, China; 3 Tongji University, Shanghai, China

**Keywords:** depleted soil, ecological risk, multi-omics, rhizosphere microbiome, sludge compost application

## Abstract

The prospective use of sludge compost for restoring depleted soils requires balancing its agronomic benefits against potential ecological risks. This study employed an integrated metagenomic and metabolomic approach to evaluate the dose-dependent effects of sludge compost on soil properties, maize growth, and rhizosphere microbial communities. Results showed that moderate compost application (≤15% w/w) enhanced soil nutrient availability, promoted root development, and enriched beneficial microbial taxa (*Streptomyces, Mesorhizobium, Flavisolibacter*), while upregulating plant stress-response metabolites (terpenoids, flavonoids). Conversely, excessive application (>20%) induced salinity stress, impaired root growth, and altered the microbial community, favoring thermophilic and xenobiotic-metabolizing taxa. Critically, high application rates led to the accumulation of residual pharmaceuticals (anti-neoplastic and anti-epileptic agents) and pesticides (insecticides and rodenticides), which correlated with the enrichment of microbial pathways associated with human diseases, highlighting a significant ecological risk. In addition, root integrity was the primary determinant of a sustainable plant-microbe feedback loop. These findings underscore the necessity for tailored application strategies to harness the soil-restorative potential of sludge compost while mitigating contaminant-driven risks, providing a framework for its safe use in sustainable agriculture.

## 1 Introduction

Resource recovery from organic waste streams is increasingly prioritized under global circular economy frameworks (Johansson, 2021; GOSC, 2024). Composting offers an effective method to transform biodegradable matter into soil amendments rich in humic substances (20–50%), microorganisms (10^7^–10^8^ CFU/g), and biostimulants (Sanchez et al., 2017; Mei et al., 2020). Application of such composts demonstrably improves soil organic matter levels (0.5–1.2% annual increase) and mitigates degradation from intensive agriculture (Zhang et al., 2021). Despite these benefits, sewage sludge-derived compost (biosolids) poses environmental risks due to inherent contaminants (pathogens, heavy metals, organic pollutants) and emerging contaminants (antibiotics, pesticides, PPCPs), now detectable at trace levels via advanced analytical techniques (Li et al., 2024). Migration and accumulation of these substances through leaching can contaminate soil, water, and potentially enter the food chain, threatening ecosystem health and food safety if environmental thresholds are exceeded.

Prior research established that biosolids are rich in nutrients, humic substances, and functional microorganisms (Mei et al., 2024). It also possesses secondary metabolites, such as indoleacetic acid and gibberellins, which function as biostimulants (Huang S. et al., 2021). These microorganisms and bioactive compounds collectively enhance plant nutrient acquisition, nutrient uptake efficiency, abiotic stress tolerance, and overall crop quality upon application to the rhizosphere. This provides a mechanistic explanation for the beneficial effects of bio-organic fertilizers on crop vegetative growth and disease suppression. Therefore, biosolids application offers a dual benefit: addressing sludge compost disposal and enhancing degraded soil productivity, thereby holding significant potential for depleted soil amelioration. Recent studies emphasize that plant–microbe interactions in the rhizosphere significantly influence contaminant degradation and nutrient cycling (Aryal, 2024a; Aryal, 2024b). Such findings underscore the importance of integrating microbial dynamics when assessing compost-induced soil restoration processes. However, the interactions (e.g., compatibility, competition, synergism) between exogenous compost-associated microorganisms and the indigenous soil microorganisms remain poorly understood. And the optimal application rates of compost and their associated environmental risks require further elucidation.

To determine the optimal application rates, the graded compost rates during seedling stage and a combined fertilization strategy at the heading stage were conducted. To characterize the interactive microorganism dynamics, this study integrates metagenomic and metabolomic analyses. These approaches characterized microbial community succession and metabolite profile shifts throughout compost production and after soil application. Microbial dynamics correlated with soil physicochemical properties (pH, EC, available N and P), while maize (*Zea mays*) growth assessments at seedling and heading stages will quantify plant responses. Key interactions involving rhizosphere microbes, root exudates, and compost-derived compounds were investigated. Additionally, non-targeted Liquid Chromatography-Mass Spectrometry (LC–MS) was analyzed considering the environmental fate of relevant beneficial and detrimental compounds such as secondary metabolites, pharmaceuticals and pesticides in treated soil. This research generated crucial insights for developing informed, sustainable strategies for utilizing biosolids in agriculture.

## 2 Materials and methods

### 2.1 Experimental design and sample collection

Sewage sludge was obtained from a municipal wastewater treatment plant located in the Southern Plains of China and processed via anaerobic digestion followed by aerobic fermentation, ensuring compliance with land application standards. Heavy metal concentrations were previously confirmed to be below permissible limits (Mei et al., 2023). Analysis revealed low concentrations of polycyclic aromatic hydrocarbons (PAHs, 0.31–0.65 mg/kg), antibiotics (0.75–0.98 mg/kg), and disinfection by-products (DBPs, 2.5–4.5 μg/kg), confirming the sludge’s suitability as a soil amendment. Experimental soil, sampled from the 0–30 cm topsoil of a depleted area, was prepared by crushing, removing debris, and sieving (3-mesh, 6.7 mm) to achieve uniformity. Baseline soil characteristics are detailed in Supplementary Table S1.

**Figure 1:**
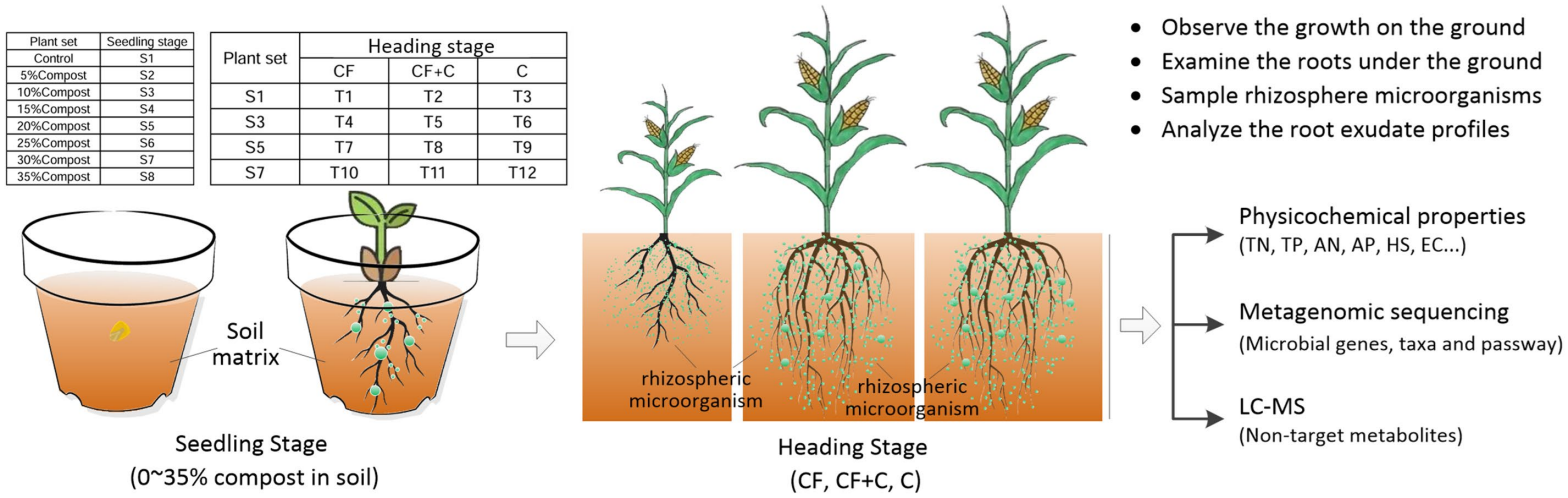
Flow chart of experimental procedures.

A pot experiment was conducted to evaluate the effects of biosolids on maize growth. Homogenized soil was mixed with compost at various mass ratios. Maize seeds, selected for uniform size and morphology, were imbibed for 48 h prior to sowing. The seedling stage comprised eight treatments with the compost application rates of 0–35% w/w, noted as S1–S8 and each with six replicates (*n* = 6). Following 30 days of growth, S1, S3, S5, S7 were selected and subjected to different fertilization strategies. To minimize plant phenotypic variation, the tallest and shortest seedlings were discarded, and three relatively uniform plants were selected for transplantation into larger pots for heading stage cultivation. The heading stage experiments consisted of three treatment groups: chemical fertilizer only (CF); a combination of chemical fertilizer and composted material (CF + C) and composted material (C), resulting in 12 experimental groups noted as T1-T12 and each with three replicates (*n* = 3). The application rates for all treatments were equivalent to 500 kg/ha. Specifically, the combined fertilization treatment involved 30% compost and 70% compound fertilizer (w/w). The compost-only application group adopted the 15% application rate consistent with the seedling stage, ensuring plant tolerance to compost. The experimental setup is depicted in Figure 1. Plant growth parameters were monitored during both stages, and rhizosphere soil samples were collected for microbial community and metabolomic analyses.

### 2.2 Plant growth assessment and soil physicochemical characterization

Maize growth was assessed at designated developmental stages by measuring key plant parameters, including stem height, stem diameter, root length and biomass. Rhizosphere soil samples were collected, air-dried, ground, sieved to remove debris prior to physicochemical analysis. Soil pH and electrical conductivity (EC) were determined potentiometrically (SFA, 1999a, 1999b). Soil organic matter (SOM) was quantified using the potassium dichromate oxidation method (MOA, 2006). Total nitrogen (TN) and available nitrogen (AN) were measured via the Kjeldahl method (MEE, 2014; SFA, 2015a). Total phosphorus (TP) and available phosphorus (AP) were determined spectrophotometrically using the molybdenum blue method following digestion (MEE, 2011; MOA, 2014). Total potassium (TK) and available potassium (AK) were analyzed by atomic absorption spectrophotometry after acid digestion (MOA, 2004; SFA, 2015b).

### 2.3 DNA extraction and metagenomic sequencing

Total genomic DNA was extracted from sludge and soil samples using the FastDNA™ Spin Kit for Soil (MP Biomedicals, Southern California, USA) in accordance with the manufacturer’s protocol. The Concentration and purity of the extracted DNA were assessed using a TBS-380 fluorometer and a NanoDrop2000 spectrophotometer, respectively. Integrity of the DNA extracts was verified via electrophoresis on a 1% agarose gel. The DNA extract was then fragmented to an average size of about 350 bp using a Covaris M220 system (Gene Company Limited, China) for the preparation of paired-end libraries. Library construction carried out with the NEXTFLEX^®^ Rapid DNA-Seq (Bioo Scientific, Austin, TX, USA), during which adapters containing the full complement of sequencing primer hybridization sites were ligated to the blunt-end fragments. Finally, paired-end sequencing was conducted on an Illumina NovaSeq platform (Illumina Inc., San Diego, CA, USA) at Majorbio Bio-Pharm Technology Co., Ltd. (Shanghai, China) employing the NovaSeq 6,000 S4 Reagent Kit v1.5 (300 cycles) according to the manufacturer’s instructions^1^. Sequence data associated with this project have been deposited in the NCBI Short Read Archive database under SRA accession number PRJNA1248781.

### 2.4 Metabolite profiling

Metabolite extraction was performed on 100 mg aliquots of compost or solid samples. Each aliquot was placed in a 2 mL centrifuge tube with a single 6 mm diameter grinding bead. Then, 800 μL of extraction solution (methanol: water = 4:1, v/v), spiked with a mix of four internal standards (each at 0.02 mg/mL, including L-2-chlorophenylalanine), was added to each tube. The samples were homogenized using a Wonbio-96c frozen tissue grinder (Shanghai Wanbo Biotechnology Co., Ltd.) at 50 Hz for 6 min while maintained at −10 °C. This was followed by low-temperature ultrasonic extraction for 30 min at 5 °C and 40 kHz. Subsequently, the extracts were incubated at −20 °C for 30 min and then centrifuged at 13,000 *g* for 15 min at 4 °C. The resulting supernatant was carefully transferred to an injection vial for subsequent LC–MS/MS analysis.

### 2.5 Statistical analyses

Soil and plant parameters were presented as mean ± standard deviation (SD) of three independent replicates. Statistical significance among treatment groups was determined by one-way analysis of variance (ANOVA) followed by *post hoc* multiple comparison tests. After confirming a significant overall P-test (*p* < 0.05), pairwise comparisons were performed. To enhance clarity and interpretability in table presentations, significant differences are denoted by letter display. All analyses were conducted using R software version 4.3.0.

Metagenomic and untargeted metabolomic data were analyzed using a multi-tiered statistical approach. Raw sequences were quality-filtered, de-hosted, assembled, and annotated to produce species and functional gene abundance profiles. Metabolomic data underwent peak extraction, retention time correction, alignment, and compound identification, yielding a metabolite intensity table. Both datasets were normalized to minimize technical and scale-related variation. Differential and multivariate analyses were performed per omics layer. Significant species and metabolites were identified using the Kruskal–Wallis test (*p* < 0.05, VIP > 2). Principal Coordinate Analysis (PCoA), Variance Inflation Factor (VIF), Redundancy Analysis (RDA), Variation Partitioning Analysis (VPA), Variable Importance in Projection (VIP), Linear Discriminant Analysis Effect Size (LEfSe) were used to identify discriminatory features. Microbial alpha diversity was assessed employing the Chao index for richness and Shannon index for diversity estimators. Also, a Pearson correlation analysis explored associations between microbial taxa, functional genes, and metabolites, enabling systematic interpretation of microbial functional activity in relation to environmental phenotypes.

## 3 Results

### 3.1 Beneficial appropriate application rates of sludge compost for the improvement of soil quality and the facilitation of seedling root growth

**Figure 2:**
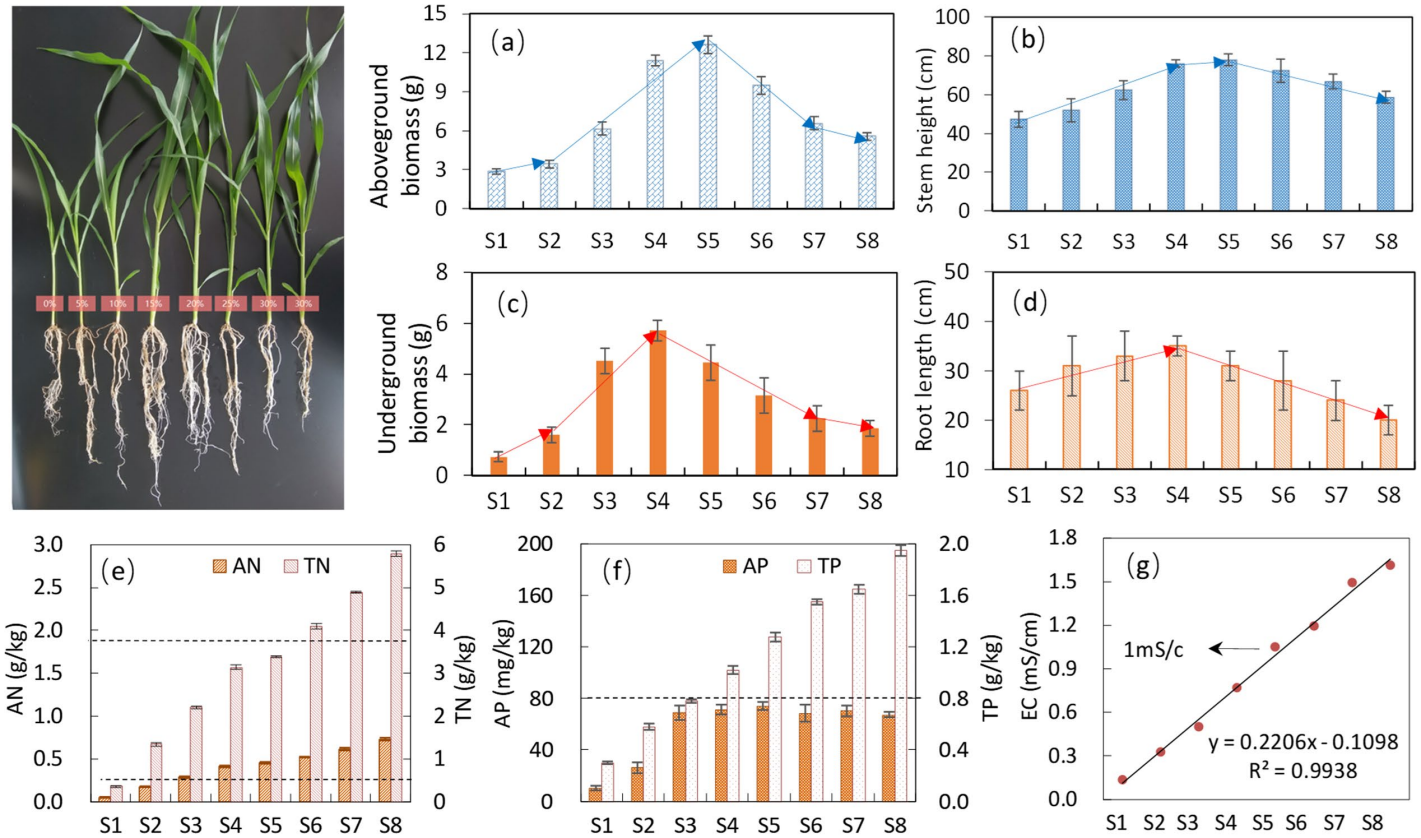
Response of maize growth and soil quality to varying rates of compost application at seedling stage. **(a)** aboveground biomass; **(b)** stem height; **(c)** underground biomass; **(d)** root length; changes in soil quality: **(e)** AN & TN; **(f)** AP & TP and **(g)** EC.

Soil amendment with sludge compost elicited differential effects on maize growth and soil properties during seedling stage. As shown in Figure 2 and Supplementary Figure S3, plant growth parameters such as aboveground biomass (Figure 2a) and stem height (Figure 2b) peaked at the 20% compost application rate (treatment S5). Conversely, parameters such as underground biomass (Figure 2c) and root length (Figure 2d) were maximized at the 15% rate (treatment S4). This suggests differential sensitivity between shoot and root systems to compost application rates, with underground components exhibiting optimal response at lower concentrations. This indicates a potential threshold for compost application regarding root development, beyond which benefits diminish or inhibitory effects may occur.

Significant alterations in soil physicochemical properties were observed across treatments (Figure 2). Compared to the control (S1), the treatment groups (S2-S8) significantly increased soil concentrations of TN & AN (Figure 2e), TP & AP (Figure 2f). A positive correlation was observed between AN and TN. Consistent with previous reports where organic nitrogen typically dominates TN (>90%) but available nitrogen constitutes a small fraction (10–15%) (Gilmour, 2021; Gao et al., 2023), observed increase in TN generally corresponded with increases in AN, suggesting enhanced nitrogen availability for plant uptake. However, referencing typical TN ranges for cultivated Chinese soil (0.4–3.8 g/kg; NATESC, 2015), Figure 2e indicates that TN contents fell below (T1, 0.36 g/kg) or potentially exceeded (T6-T8, >4 g/kg) this optimal range in some treatments.

Regarding phosphorus, essential for cellular processes and particularly crucial for early root development (Ziadi et al., 2013), increased TP did not consistently translate to higher AP. Our analysis showed AP comprised <10% (10–80 mg/kg) of TP across all treatments (Figure 2f). Beyond a TP threshold of approximately 0.8 g/kg (Figure 2f, horizontal line), further compost addition did not elevate AP level. Notably, soil EC (Figure 2g), an indicator of salinity, increased linearly with compost application rate. Referencing horticultural substrates standards where optimal EC is typical <1.0 mS/cm (SILASP et al., 2016), compost rates ≥20% (T6-T8) resulted in EC values exceeding this threshold, indicating potential salinity stress. Combined with the growth data, these findings suggest that root development is particularly sensitive to compost-induced soil physicochemical changes, including potential salinity effects, which aligns with previous research on salinity impacts on crop growth (Liu et al., 2016). While aboveground growth improved up to 20–25% compost rates, root inhibition was apparent at rates above 15%. Considering the root system’s critical role in water and nutrient uptake, these findings suggest maintaining compost application below 15% during the seedling stage is advisable for optimal root establishment.

Heading stage results were presented in Table 1 and Supplementary Table S2, where bold letters mean statistical differences. Comparison across treatments indicated that compost amendments generally enhanced maize aboveground biomass accumulation compared to chemical fertilizer (CF) alone. Compared to compost only (C), the combined treatment (CF + C) yielded slightly superior biomass and was more favorable for root growth. Longitudinal comparison between seedling and heading stages highlighted the persistent impact of early root development. Specifically, treatment S7, which exhibited root impairment during the seedling stage, displayed continued growth inhibition at the heading stage. This underscores the importance of seedling root health for subsequent plant performance. Changes in soil quality during the heading stage (Tables 1e–h), particularly N and P concentrations, was consistent with the plant growth performance, indicating that soil quality is closely related to maize growth status.

**Table 1:** Response of maize growth and soil quality to varying rates of compost application at heading stage.

Samplename	CF	CF + C	C
	(a) Aboveground biomass (g)		
S1	0.00**a**	3.18 ± 0.25**b**	1.27 ± 0.33**b**
S3	6.45 ± 0.32**b**	38.18 ± 3.33**d**	28.21 ± 3.10**d**
S5	11.55 ± 0.75**c**	34.39 ± 2.50**d**	34.63 ± 2.45**d**
S7	10.58 ± 1.55**c**	30.86 ± 3.64**d**	27.39 ± 3.21**d**

Samplename	(b) Stem height (cm)		
S1	0.00**a**	46.75 ± 4.50**b**	41.5 ± 6.30**b**
S3	50.00 ± 3.50**b**	108.75 ± 5.90**d**	102.35 ± 9.10**d**
S5	62.00 ± 5.10**c**	112.00 ± 8.55**d**	101.50 ± 4.60**d**
S7	58.50 ± 3.20**c**	98.35 ± 7.20**d**	90.85 ± 7.10**d**

Samplename	(c) Underground biomass (g)		
S1	0.00**a**	0.52 ± 0.14**b**	0.51 ± 0.11**b**
S3	2.63 ± 0.34**c**	5.56 ± 1.33**d**	5.12 ± 0.45**d**
S5	2.65 ± 0.41**c**	4.72 ± 0.75**d**	3.73 ± 0.32**d**
S7	2.70 ± 0.22**c**	4.18 ± 0.88**d**	3.00 ± 0.97**c**

Samplename	(d) Stem diameter (cm)		
S1	0.00**a**	0.60 ± 0.09**b**	0.50 ± 0.00**b**
S3	0.70 ± 0.11**b**	1.30 ± 0.05**c**	1.25 ± 0.03**c**
S5	0.70 ± 0.15**b**	1.35 ± 0.00**c**	1.25 ± 0.60**c**
S7	0.80 ± 0.10**b**	1.35 ± 0.10**c**	1.20 ± 0.20**c**

Samplename	(e) TN contents in soil (g/kg)		
S1	0.33 ± 0.11**a**	2.43 ± 0.22**d**	2.14 ± 0.18**c**
S3	0.58 ± 0.13**a**	2.90 ± 0.34**d**	1.95 ± 0.24**c**
S5	1.75 ± 0.31**b**	2.77 ± 0.11**d**	1.72 ± 0.34**b**
S7	1.67 ± 0.21**b**	2.48 ± 0.45**d**	1.63 ± 0.10**b**

Samplename	(f) AN contents in soil (g/kg)		
S1	0.05 ± 0.01**a**	0.29 ± 0.03**c**	0.20 ± 0.01**b**
S3	0.05 ± 0.01**a**	0.33 ± 0.02**c**	0.25 ± 0.00**c**
S5	0.04 ± 0.00**a**	0.33 ± 0.05**c**	0.18 ± 0.03**b**
S7	0.04 ± 0.01**a**	0.23 ± 0.02**b**	0.14 ± 0.02**b**

Samplename	(g) TP contents in soil (g/kg)		
S1	0.25 ± 0.08**a**	0.71 ± 0.09**c**	0.65 ± 0.08**b**
S3	0.28 ± 0.15**a**	0.80 ± 0.15**c**	0.66 ± 0.06**b**
S5	0.27 ± 0.10**a**	0.75 ± 0.21**c**	0.72 ± 0.09**c**
S7	0.30 ± 0.12**a**	0.77 ± 0.05**c**	0.71 ± 0.05**c**

Sample name	(h) AP contents in soil (g/kg)		
S1	0.006 ± 0.000**a**	0.033 ± 0.005**b**	0.034 ± 0.005**b**
S3	0.008 ± 0.002**a**	0.038 ± 0.007**b**	0.031 ± 0.004**b**
S5	0.006 ± 0.001**a**	0.035 ± 0.010**b**	0.031 ± 0.003**b**
S7	0.004 ± 0.000**a**	0.032 ± 0.006**b**	0.026 ± 0.005**b**

The letters are statistical significance markers. Same letter means no significant difference (*p* > 0.05), while different letters indicate a significant difference (*p* < 0.05).

Based on these findings, employing a combined fertilization strategy incorporating both sludge compost (at rates optimized for seedling root health, ≤15%) and supplementary chemical fertilizer appears optimal. This approach leverages the soil conditioning benefits of compost while ensuring adequate nutrient supply, promoting root growth, enhancing nutrient availability, and ultimately supporting vegetative growth and biomass accumulation throughout the maize life cycle.

### 3.2 Restructured soil microbial community and enhanced plant tolerance to environmental stress by excessive application of sludge compost

**Figure 3:**
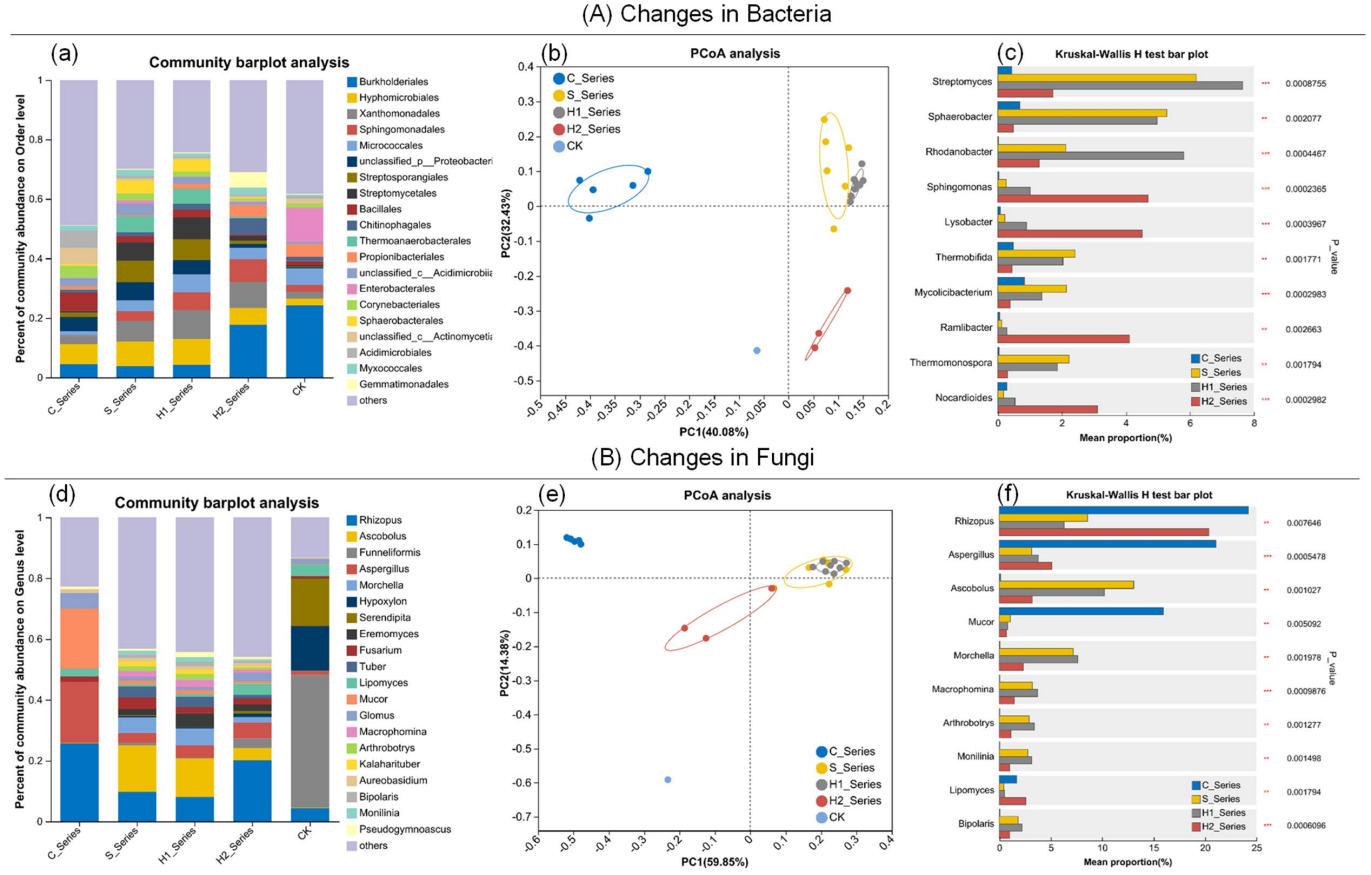
Comparative analysis of rhizosphere microbial communities across different groups using metagenomic sequencing data. **(a)** bacterial taxonomic composition at the order level; **(b)** PCoA based on Bray-Curtis dissimilarities of bacterial communities; **(c)** identification of significantly different bacterial taxa among multiple groups; **(d)** fungal taxonomic composition at the genus level; **(e)** PCoA based on Bray-Curtis dissimilarities of fungal communities and **(f)** identification of significantly different fungal taxa among multiple groups.

Alterations in soil quality and crop vegetative growth are understood to be intricately linked to the dynamics of resident soil microbial communities. To systematically investigate the adaptation and persistence of composted-derived microorganisms within the soil environment, this study employed metagenomic sequencing of rhizosphere soil samples collected during the seedling and heading stages. PCoA based on Bray-Curtis dissimilarities of bacterial community structure at the order level (Figure 3b) was performed to evaluate compositional similarity among treatment groups. The analysis revealed that the compost source samples (C1-C5, hereafter C_Series) exhibited the greatest compositional dissimilarity relative to all other groups. Notably, cured compost product (C6) demonstrated greater compositional similarity to the seedling-stage rhizosphere microbiome samples (S2-S6, hereafter S_Series). This observation suggests that compost curing process may enhance the environmental adaptability of its constituent microorganisms to the soil rhizosphere. Furthermore, the seedling-stage rhizosphere microbiomes (S_Series) displayed high compositional similarity to a subset of the heading-stage microbiome groups (H2-H9, hereafter H1_Series). In contrast, another subset of heading-stage samples (H10-H12, hereafter H2_Series) was markedly dissimilar to the H1_Series, instead exhibiting greater similarity to the background soil (S1, designated CK). Subsequent investigation revealed a correlation between the H2_Series samples and observations of severe root system damage. These findings underscore the integral connection between soil microbial diversity dynamics, consequent alterations in soil quality, and plant root health. Congruent findings emerged from the analysis of fungal communities. PCoA at the genus level (Figure 3e) indicated the most pronounced compositional dissimilarity between the C_Series and CK groups. Relatively minor differences were observed between the S_Series and H1_Series fungal communities, consistent with the patterns identified for the bacterial consortia.

Based on these established groupings, microbial alpha diversity was assessed using richness (Chao index) and diversity (Shannon index) estimators (Supplementary Figure S4). Relative to the background soil (CK), the application of composted material resulted in an increase in both the estimated richness and diversity of soil microorganisms. This effect was particularly pronounced for the fungal communities. Taxonomic profiling at the domain and phylum levels (Supplementary Figure S5) indicated bacterial dominance (relative abundance>97%) across all samples except for the CK control. In contrast, the CK sample exhibited a lower bacterial relative abundance (64.19%), with the domain Eukaryota accounting for 35.65%. Within the eukaryotic fraction of the CK sample, Streptophyta constituted the vast majority (99.99%). Notably, Fungi comprised less than 0.2% of the total microbial community sequence reads across all samples. However, despite this low relative abundance, fungi are recognized for potentially significant ecological roles, including plant pathogenesis. At the order and genus levels (Figures 3a,d), the application of compost was associated with an increase in the number of detected bacterial taxa, whereas the number of detected fungal taxa decreased compared to the CK group.

Furthermore, significant differences in microbial community composition at the genus level were evident between the control and the compost-amended experimental groups. Dominant bacterial genera within the CK group included *Acidovorax, Escherichia, Acinetobacter, Salmonella, Burkholderia,* and *Pseudarthrobacter*. The former five genera encompass species known to be plant or animal pathogens, or opportunistic pathogens (Mead et al., 1999; Zhang et al., 2013; Rhodes and Schweizer, 2016; Li et al., 2022; Zhu et al., 2023), potentially contributing to plant diseases or posing broader environmental health concerns. Conversely, dominant fungal genera identified in the CK group included *Funneliformis, Hypoxylon, Serendipita, Rhizopus,* and *Lipomyces*. These fungal genera are widely reported as beneficial, capable of establishing symbiotic relationships with plant roots or rhizosphere bacteria, thereby facilitating soil nutrient acquisition and potentially contributing to disease suppression (Onofri et al., 1999; Sefloo et al., 2019; Giovannini et al., 2020; Probst et al., 2023). Specifically, *Funneliformis* belongs to the arbuscular mycorrhizal fungi (AMF), which form arbuscular and reticulate structures within plant roots, enhancing the absorption of water and minerals from the soil. This symbiosis, in turn, can improve plant resilience to various abiotic stresses such as drought, salinity, and pollution, as well as biotic stresses induced by other organisms. Additionally, AMF are known producer of glomalin, a glycoprotein recognized for its role in enhancing soil aggregate stability (Onyeaka et al., 2024).

**Figure 4:**
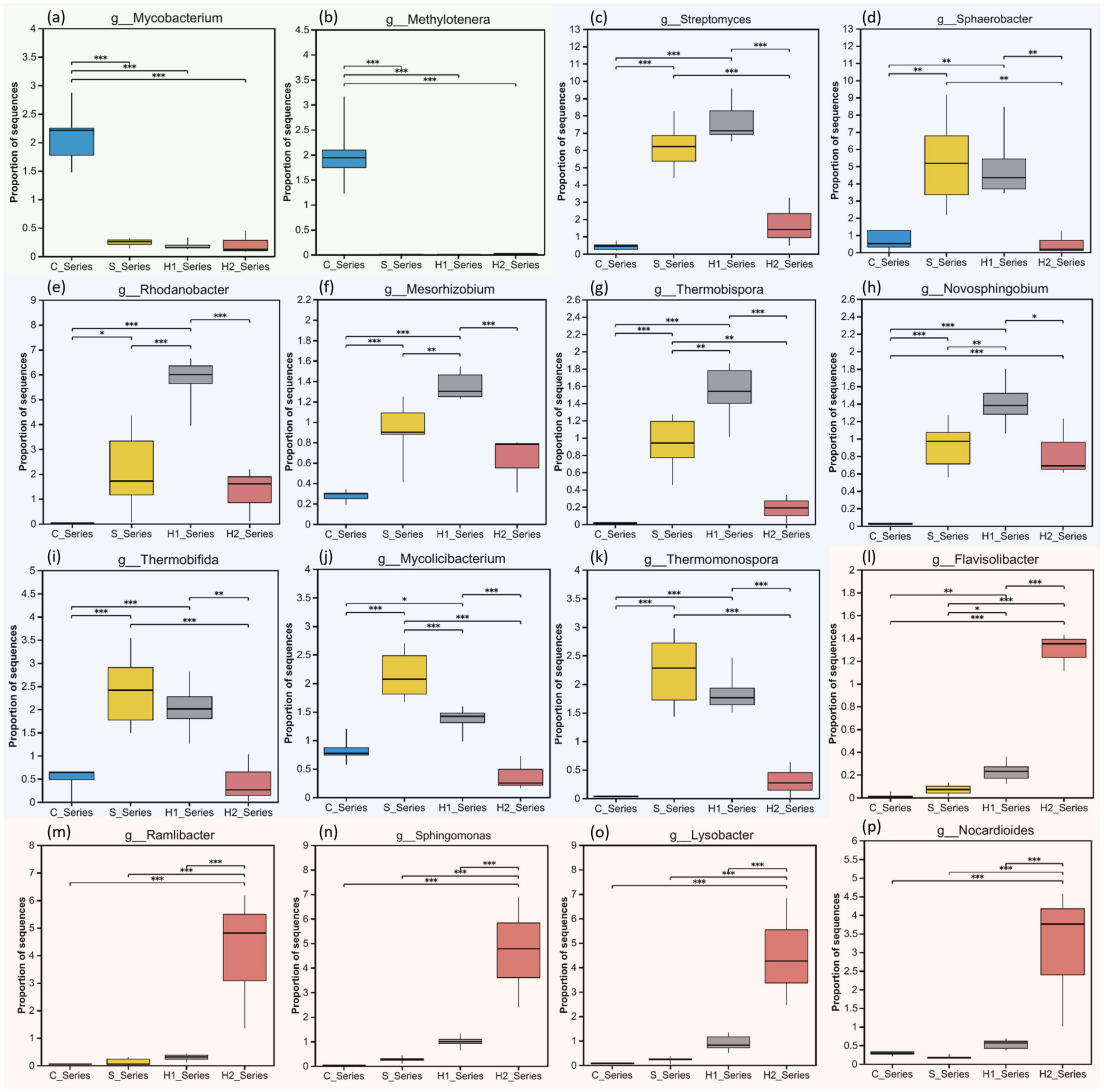
Differentially abundant bacterial taxa across multiple groups (*p* < 0.05). **(a–b)** Dominant bacterial taxa characteristic of the composting process. **(c–k)** Dominant bacterial taxa characteristic of the seedling and heading stages. **(l–p)** Dominant bacterial taxa unique to the root-damaged experimental groups. (*p < 0.05, **p < 0.01, ***p < 0.001).

To identify microbial taxa exhibiting significant differences in abundance across experimental groups, the non-parametric Kruskal-Wallis sum-rank test was employed (*p* < 0.05). This analysis highlighted differentially abundant bacterial and fungal taxa between treatment groups and developmental stages (Figures 3c,f, 4). Significantly different bacterial taxa were categorized based on their predominant associations: (1) Taxa characteristic of the compost source material (Figure 4a,b), such as *Mycobacterium* and *Methylotenera*, genera implicated in organic matter transformation or degradation (Haritash and Kaushik, 2009; Liu et al., 2021). (2) Taxa predominantly abundant during seedling and heading stages (Figures 4c–k), which encompassed several genera from Actinobacteria (*Streptomyces, Thermobispora, Thermobifida, Thermonospora, Mycolicibacterium*), Proteobacteria (*Rhodanobacter, Mesorhizobium*, *Novosphingobium*), and Chloroflexi (*Sphaerobacter*). The enrichment of Actinobacteria and Proteobacteria in compost-treated soils is consistent with their known roles in organic pollutant degradation and rhizosphere resilience. For example, the rise in *Streptomyces* a genus linked to xenobiotic degradation and antibiotic production that suggests a functional shift toward detoxification and biocontrol (Xie et al., 2010; Barka et al., 2016). Thermophilic genera such as *Thermobispora, Thermobifida, Thermonospora,* and *Sphaerobacter* are established degraders of complex organic matter often found in compost (Luo et al., 2024). Similarly, *Rhodanobacter* and *Mesorhizobium* participate in nitrogen cycling (Brígido et al., 2017; Tao et al., 2022), whereas *Mycolicibacterium* and *Novosphingobium* contribute to pollutant breakdown and bioremediation (Liu et al., 2005; Golubev et al., 2021). (3) Taxa significantly enriched in the root-damaged experimental groups (H2_Series, Figures 4l~p), including *Flavisolibacter* (Bacteroidetes), *Ramlibacter, Sphingomonas, Lysobacter* (Proteobacteria), and *Nocardioides* (Actinobacteria). *Flavisolibacter* species are recognized as plant growth-promoting rhizobacteria (PGPR), capable of effective rhizosphere colonization and antagonism against phytopathogens (Zhang et al., 2024). *Lysobacter* species are established biocontrol agents against soilborne pathogens (Fu et al., 2018), while *Sphingomonas,* and *Nocardioides* have documented roles in pollutant degradation and bioremediation (Leung et al., 1999; Ma et al., 2023). Collectively, the observed enrichment patterns especially the selection for taxa involved in pollutant degradation and those with plant-beneficial traits (PGPR, biocontrol) in root-damaged groups; point to a microbial community response to stress linked to poor root development. These taxonomic shifts likely reflect functional adaptation in pathways related to xenobiotic degradation and rhizosphere detoxification, representing a compensatory ecological mechanism within the rhizosphere microbiome.

**Figure 5:**
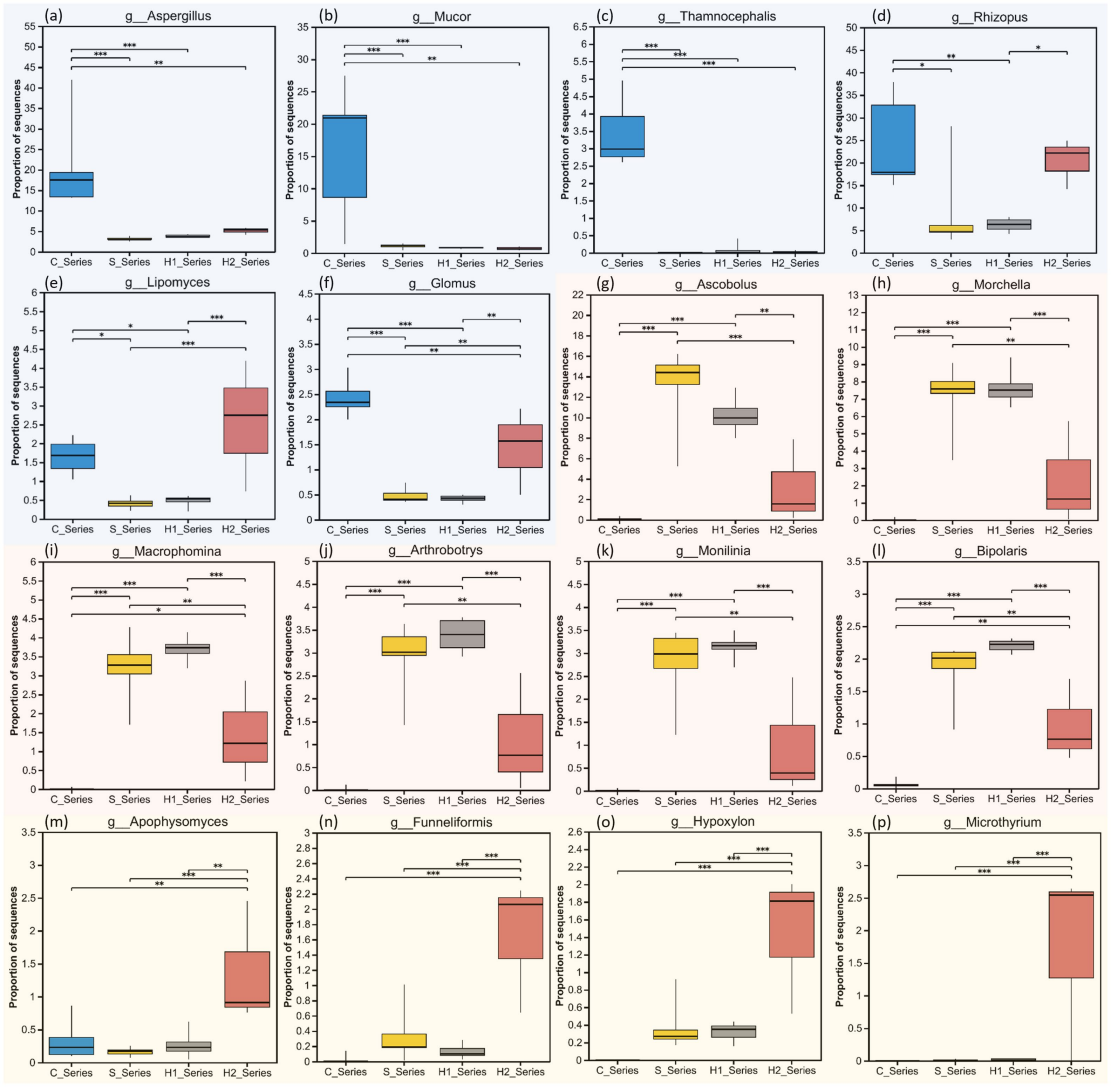
Differentially abundant fungal taxa across multiple groups (*p* < 0.05). **(a–b)** Dominant fungal taxa characteristic of the composting process. **(c–k)** Dominant fungal taxa characteristic of the seedling and heading stages. **(l–p)** Dominant fungal taxa unique to the root-damaged experimental groups. (*p < 0.05, **p < 0.01, ***p < 0.001).

Analysis identified significantly different fungal taxa among the experimental groups (Figure 5). These taxa were categorized based on their predominant associations: (1) Taxa characteristic of the compost source material (Figures 5a,b), including *Rhizopus, Aspergillus, Mucor, Lypomyces, Thamnocephalis,* and *Glomus*. The former four genera are recognized for their roles in fermentation and the decomposition of complex organic matters. *Thamnocephalis,* frequently isolated from animal manure (Tanabe et al., 2000), likely originated from the compost. *Glomus,* a genus of AMF, is known for promoting plant growth, improving soil structure, and enhancing crop yield and stress resistance (Wang et al., 2008). Considering the prior identification of *Funneliformis*, another AMF genus in CK, it can be hypothesized that AMF colonization may be favored under conditions of environmental extremes (either nutrient-poor or nutrient-rich). (2) Taxa predominantly abundant during seedling and heading stages (Figures 5c–k), which comprised Ascomycota genera (*Ascobolus, Morchella, Arthrobotrys, Macrophomina, Monilinia,* and *Bipolaris*). *Ascobolus, Morchella* and *Arthrobotrys* are reported as beneficial soil fungi, with some species exhibiting biocontrol capabilities (Zhang et al., 2018; Zhang et al., 2022; Manici et al., 2024). In contrast, genera such as *Macrophomina, Monilinia,* and *Bipolaris* include species recognized soilborne or plant pathogens, which under conducive conditions can cause diseases like root rot, stem rot, and leaf blight (Gayed, 1963; Peng et al., 2016; Liu et al., 2018). It should be noted that their detection here, particularly in the control (CK) or high-dose groups, occurred at low relative abundances and was not associated with visible disease symptoms. This underscores the context-dependent nature of fungal pathogenicity, which varies according to specific strain, abundance, environmental conditions, and host health status. (3) Taxa significantly enriched in the root-damaged experimental groups (H2_Series, Figures 5l–p), including *Apophysomyces, Funneliformis, Microthyrium,* and *Hypoxylon*. These genera are largely reported as beneficial. *Apophysomyces, Funneliformis,* and *Microthyrium* have been linked to alleviating plant salt stress and remediating saline-alkali soils through the secretion phytohormones or antioxidant (Giovannini et al., 2020; Li et al., 2020). *Hypoxylon* species are known to exhibit pronounced antagonistic activity against certain plant pathogenic fungi (Sanchez-Fernandez et al., 2020). Collectively, the enrichment of these potentially beneficial fungal taxa – involved in stress mitigation or pathogen antagonism – within the groups exhibiting poor root health is notable. This pattern, mirroring observations in the bacterial community, suggests a complex microbial response dynamic wherein specific functional guilds may be selected for under conditions adverse to plant root development, possibly representing a compensatory or adaptive shift within the rhizosphere microbiome.

### 3.3 Available phosphorus and plant root systems: critical environmental determinants of soil microbial biomass, activity and community composition

To identify key environmental factors shaping soil microbial community composition, we performed integrated analyses using microbial community data and measured environmental variables. Multicollinearity among potential explanatory variables was first assessed using VIF analysis. For the seedling stage, initial candidate factors included TN, TP, AN, AP, and EC, as detailed in Supplementary Table S3. VIF analysis revealed that only AP and EC exhibited values below 10, and were thus retained for subsequent analyses (Supplementary Table S4). For the heading stage, the initial set of factors comprised Root condition (Root), TN, TP, TK, AN, AP, and AK, as detailed in Supplementary Table S5. Following VIF screening, Root, TN, TP, and TK were identified as suitable non-collinear variables (VIF < 10) for inclusion in further analyses (Supplementary Table S6).

**Figure 6:**
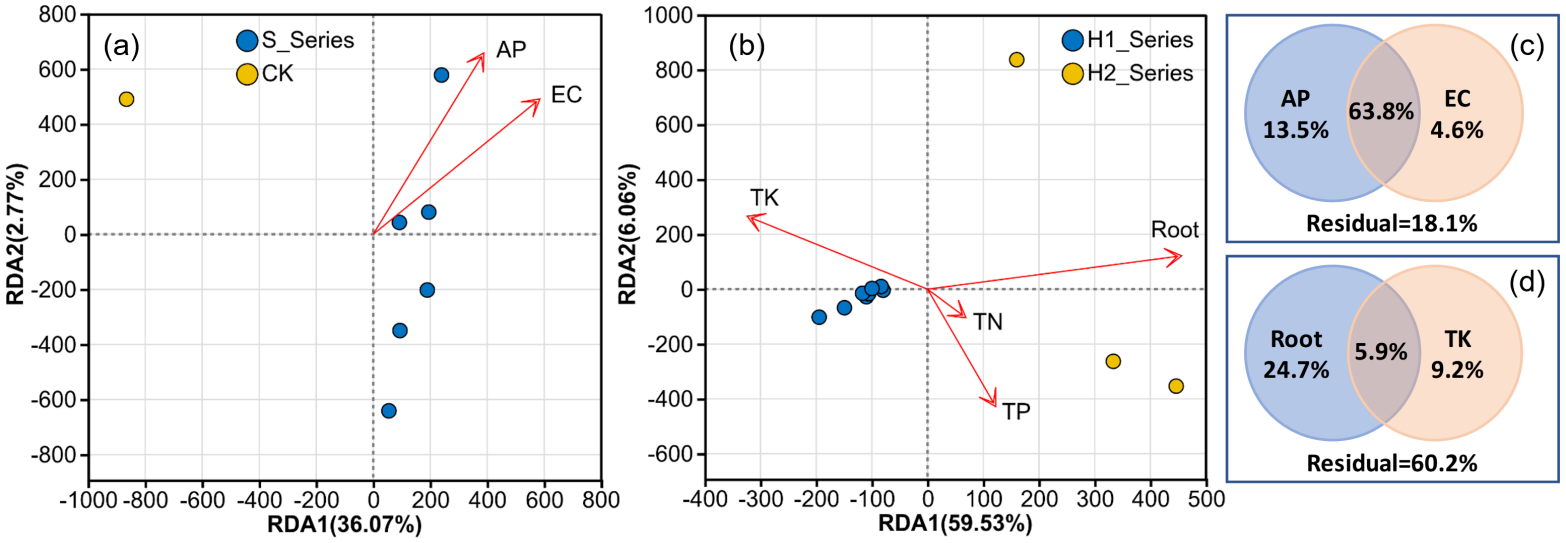
Relationships between microbial taxa and environmental factors. **(a)** RDA analysis of seedling stage; **(b)** RDA analysis of heading stage; **(c)** VPA analysis of seedling stage and **(d)** VPA analysis of heading stage.

RDA was subsequently conducted to elucidate the relationships between microbial community structure and the selected environmental factors (Figure 6). At the seedling stage (Figure 6a), RDA revealed significant association between microbial community ordination and both AP and EC. Samples corresponding to higher compost application rates (S5-S6) tended to cluster along gradients positively correlated with these factors. At the heading stage (Figure 6b), the Root variable (reflecting root system damage, potentially linked to initial compost treatments) emerged as the strongest explanatory factor for variation in microbial community structure, followed by TK. A negative correlation between the Root variable and TK levels was also observed, consistent with literature indicating potassium’s role as enzymatic activator crucial for metabolic processes and photosynthetic translocation, potentially compromised by poor root health (Shin, 2014).

To quantify the explanatory power of these key factors, VPA was performed. At the seedling stage (Figure 6c), AP independently explained 13.5% of the microbial community variance, while EC explained 4.6%, identifying available phosphorus as the primary environmental correlate of microbial structure during early growth, followed by salinity. At the heading stage (Figure 6d), the Root variable explained 24.7% of community variance, and TK explained 9.2%. This underscores the crucial influence of root system development and health in shaping the rhizosphere microbiome and influencing nutrient dynamics (specifically potassium) later in the plant life cycle. These findings suggest a model wherein sufficient phosphorus availability during the seedling stage may promote robust root establishment. A well-developed root system subsequently enhances plant access to soil resources, influencing both overall plant performance and the associated microbial community structure, particularly concerning potassium availability during the heading stage.

### 3.4 Effect of sludge compost impacts on plant secondary metabolism, composition and activity of plant-associated microbiome, acting on the crop nutritional growth and tolerance to environmental stress

To elucidate the biochemical mechanisms linking compost-derived microbial shifts to alteration in plant–soil interactions, this study employed an integrated multi-omics approach combining soil metagenomics and non-targeted metabolomics. The primary objective was to identify correlations between changes in microbial community composition and soil metabolite profiles. Initially, metabolites exhibiting significant differential abundance across treatment groups were identified using the non-parametric Kruskal-Wallis sum-rank test (*p* < 0.05) alongside VIP scores>2.0 (derived from the multivariate analysis and PLS-DA). This process yielded 162 significantly differential metabolites (Supplementary Table S7). Based on their functions, these metabolites were classified into several major categories: plant secondary metabolites (*n* = 48), pharmaceuticals and pesticides (*n* = 40), sugar alcohols and amino acids (*n* = 34), organic acids and lipids (*n* = 18), microbial secondary metabolites (*n* = 7), vitamins (*n* = 5), and others (*n* = 10). Given their established relevance to soil health, ecological function, and potential risk assessment, plant secondary metabolites, pharmaceuticals and pesticides were prioritized for subsequent detailed analyses.

**Figure 7:**
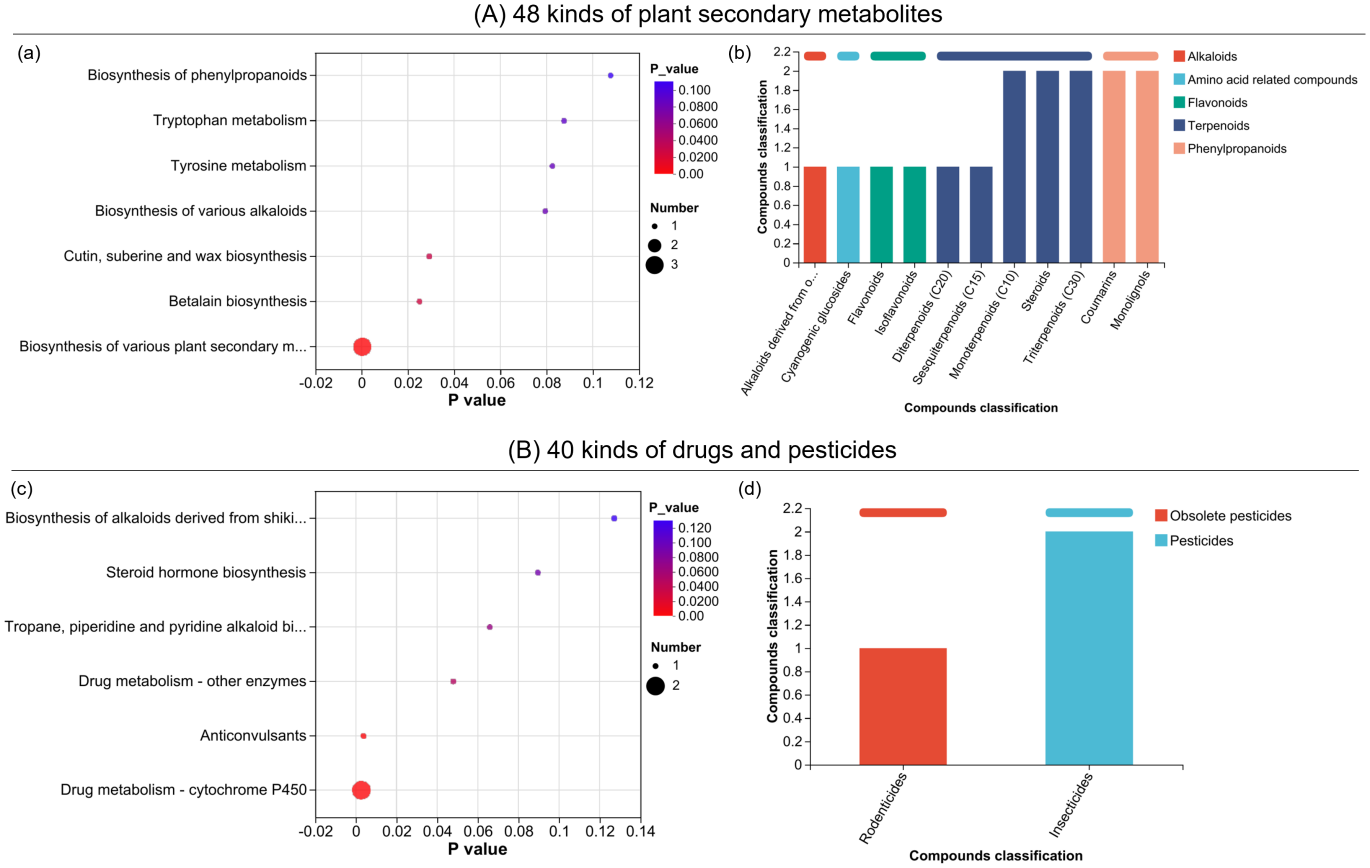
KEGG compound classification and pathway enrichment analysis of metabolites across multiple groups. **(A)** plant secondary metabolites and **(B)** pharmaceuticals and pesticides.

**Figure 8:**
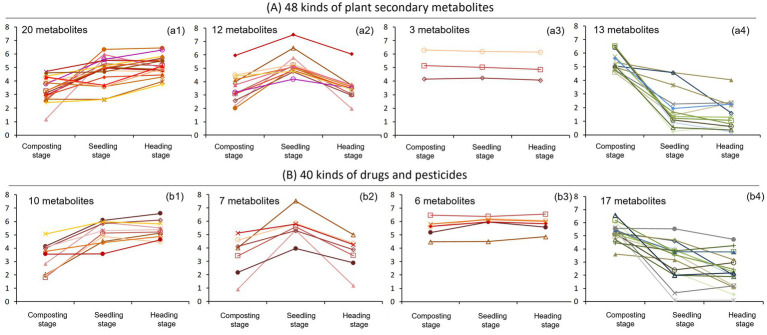
Changing trends of metabolites throughout different stages. **(A)** plant secondary metabolites and **(B)** pharmaceuticals and pesticides.

Focusing first on plant secondary metabolites, KEGG pathways enrichment analysis revealed a significant over-representation of pathways related to the biosynthesis of various plant secondary metabolites (Figure 7a). Analysis of the abundance profiles for the 48 differentially abundant plant secondary metabolites allowed for further subclassification (Figure 8a). Notably, 32 metabolites generally increased in abundance with compost application rate, although 12 of these showed reduced abundance in samples associated with root damage. Conversely, 13 metabolites, likely originating from the compost material itself, decreased in abundance following soil amendment, potentially reflecting microbial degradation of transformation processes. KEGG compound classification (Figure 7b and Supplementary Table S8) indicated that these differential plant secondary metabolites primarily included terpenoids (*n* = 14), flavonoids (*n* = 5), phenylpropanoids (*n* = 4), glycosides (*n* = 9), alkaloids (*n* = 5), and others (*n* = 11). Terpenoids compounds include important phytohormones regulating plant growth and development, and signaling molecules involved in defense responses and environmental stress protection (Huang Y. et al., 2021). Flavonoids compounds are known for their antioxidant properties, scavenging reactive oxygen species within plant (Koes et al., 1994). Structural modifications, such as glycosylation and hydroxylation, can further diversify their pharmacological activities (Zhan et al., 2019; Ren et al., 2022). Phenylpropanoids compounds serve as precursors for phytohormone biosynthesis or regulate phytohormone metabolism and signaling, also crucial for lignin biosynthesis involved in cell differentiation and tissue formation (Kurkin, 2003). Glycosides compounds function as structural components of plant cell walls and intermediates in energy storage and transport within plants (Dembitsky, 2004). Alkaloids compounds are implicated in phytohormone metabolism and the regulation of plant growth processes (Kries and O'Connor, 2018).

**Figure 9:**
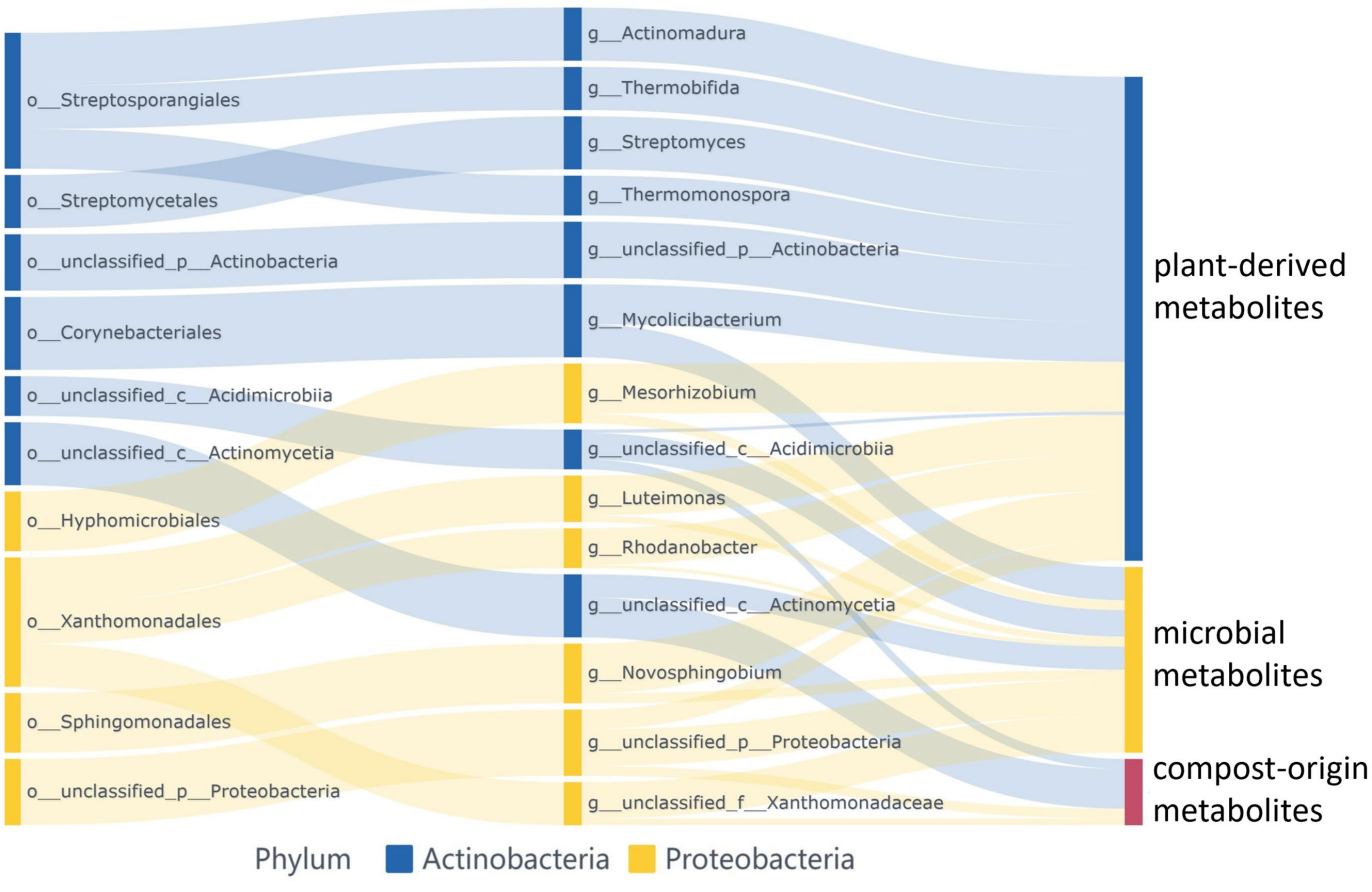
Correlation analysis of microbial taxa and plant secondary metabolite abundance.

Differential metabolite abundance patterns across treatment groups were detailed in Figure S6. Following compost amendment, 20 specific metabolites exhibited significant upregulation in maize rhizosphere during both seedling and heading stages, which were identified as plant-derived metabolites. Concurrently, 13 metabolites showed significant downregulation, which likely originated from the compost itself and decreased in abundance after entering the soil. These were categorized as compost-origin metabolites. Additionally, 15 metabolites displayed fluctuating trends, which may be associated with microbial dynamics and were thus recognized as microbial metabolites. Based on the grouping, correlation analyses were performed between metabolite categories and microbial species, with the results presented in Supplementary Figure S7 and Figure 9. Results indicated that bacterial taxa within the phylum Actinobacteria displayed the strongest overall association, followed by Proteobacteria. Plant-derived metabolites are mainly regulated by species belonging to the phylum Actinobacteria. At the genus level, key positive associations were identified with *Mycolicibacterium, Streptomyces, Thermomonospora, Thermobifida* and *Actinomadura*. Notably, the former four were also dominant, functionally relevant genera within the compost material. As discussed previously, *Streptomyces* produces diverse bioactive antimicrobial metabolites (Zhang et al., 2013; Zhu et al., 2023). *Mycolicibacterium* participates in bioremediation, including polycyclic aromatic hydrocarbons (PAHs) degradation (Golubev et al., 2021), and *Thermomonospora* and *Thermobifida* are thermophilic degraders of recalcitrant organic matter (Liu et al., 2023). These facts provide further corroboration that the upregulation of plant-derived metabolites aligns with known phytoremediation responses to environmental toxicants (Zhang et al., 2025). Overall, these functional bacteria introduced via compost application likely stimulated the synthesis and accumulation of plant-derived metabolites. This process subsequently contributed to the detoxification of environmental contaminants and played a regulatory role in enhancing crop stress resilience.

Conversely, in groups with significant root damage (H2_Series), the abundance of most plant secondary metabolites decreased, concomitant with reduced microbial abundance (Supplementary Figure S6). However, two specific terpenoids, ingenol and 13-hydroxy-7,14-labdadien-6-one, exhibited significant upregulation in these H2_Series samples. Both compounds have been reported anti-inflammatory activities and potential roles in plant defense against pathogens and environmental stressors (Bergman et al., 2019). Further correlation analyses exploring associations with broader microbial domains (Supplementary Figures S8, S9) suggested that the abundance of these two terpenoids correlated more strongly with eukaryotic taxa (Streptophyta and Chordata).

### 3.5 Potential threat to soil health with sludge compost to the enrichment of pharmaceutical and pesticide residues

**Figure 10:**
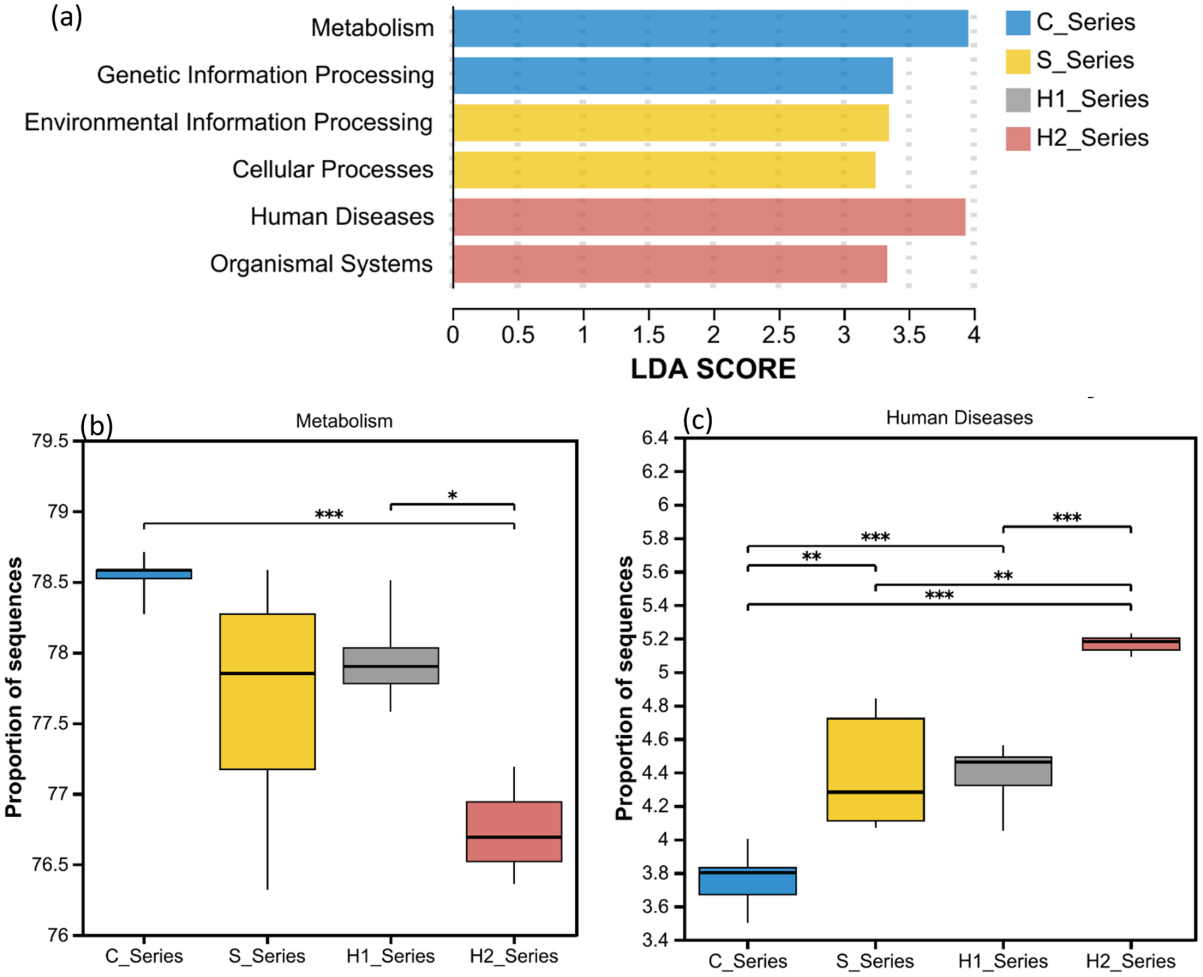
Differential analysis of metabolic pathways across treatment groups. **(a)** LEfSe analysis and **(b–c)** differential functional analysis with Kruskal-Wallis test. (*p < 0.05, **p < 0.01, ***p < 0.001).

To evaluate potential ecological risks associated with compost application, the differential abundance of microbial functions (annotated via KEGG Orthology) across treatment groups was assessed using the non-parametric Kruskal-Wallis tests. Subsequently, LEfSe analysis was employed to identify functions exhibiting significant discriminatory power between groups (Figure 10a). Differential functional analysis with Kruskal-Wallis test indicated that functions classified under “Metabolism” were significantly enriched (*p* < 0.05) in the compost source material compared to soil samples (Figure 10b), reflecting elevated microbial metabolic activity characteristic of composting process. Conversely, functions categorized under “Human Diseases” displayed significantly enrichment (*p* < 0.05) specifically in the root-damage treatment groups (H2_Series, Figure 10c). In particular, “Drug resistance: antineoplastic” emerged as the predominant metabolic pathway within the “Human Disease” category according to KEEG pathway analysis (Supplementary Figure S10). This finding suggests that environmental conditions or microbial community shifts linked to impaired root health may be associated with an increase in potential ecological risk indicators.

Complementary non-targeted metabolomics analysis identified 40 metabolites, putatively classified as pharmaceuticals (*n* = 34) or pesticides (*n* = 6), exhibiting differential abundance (*p* < 0.05 and VIP > 2.0) across treatment groups (Supplementary Table S9). The KEGG pathway primarily associated with these metabolites showed significant enrichment in “Drug metabolism - cytochrome P450” (Figure 7c). Based on their abundance profiles (Figure 8b), 10 of these compounds increased following compost application, whereas 17 compounds decreased, the latter potentially indicating microbial degradation in the amended soil. The remaining 13 compounds displayed varied trends profiles across treatment groups.

KEGG compound classification (Figure 7d) indicated that the differentially abundant pesticides primarily comprised insecticides and rodenticides. Given that no pesticides were applied during the experimental period, these compounds are presumed to represent legacy contaminants present in the experimental soil. Among the 34 differentially abundant pharmaceuticals, anti-neoplastic and anti-epileptic agents constituted the predominant classes. This suggests a likely origin pathway involving human excretion into wastewater, subsequent concentration in sewage biosolids potentially used in compost production, and persistence in the soil following amendment.

We acknowledge that the present study lacks quantitative contaminant measurements, which limits a direct assessment of absolute environmental concentrations. Nevertheless, the enrichment of risk-related metabolic pathway (e.g., “Drug resistance: antineoplastic”) and the detection of pharmaceutical and pesticide residues serve as relative indicators of contaminant presence and potential bioavailability. These functional and metabolite shifts suggest a plausible link between compost amendment and the emergence of contaminant-associated ecological signatures, underscoring the need for further investigation into the ecotoxicological effects of these compounds on soil microorganisms and ecosystem biodiversity.

## 4 Discussion

This study shows that sludge compost application produces dose-dependent effects on agroecosystem function, defining a threshold between synergistic benefit and systemic stress. Roots and shoots differ in sensitivity, with root integrity emerging as the primary determinant of sustainable application. Specifically, root inhibition at doses exceeding 15% (w/w), which correlated with increased soil salinity (EC > 1.0 mS/cm), defines a critical physicochemical constraint. Early-stage root impairment produced a legacy effect that reduced subsequent plant performance and restructured the rhizosphere microbiome, indicating that root condition predominantly drives microbial community assembly during later growth stages.

Integrative analysis reveals that moderate application rates (≤15%) enhance phosphorus and nitrogen availability, facilitate root establishment and enrich the rhizosphere with beneficial taxa such as *Streptomyces, Mesorhizobium* and *Flavisolibacter*. Metabolomic corroborates these effects, showing upregulation of stress-responsive terpenoids and flavonoids. Strong correlation between these metabolites and key bacterial phyla (Actinobacteria and Proteobacteria) suggest a positive feedback loop in which improved soil chemistry promotes root-mediated microbiome assembly that, in turn, enhances plant metabolic defenses.

Conversely, excessive application (>20%) disrupts this synergy by introducing dual stressors: salinity and organic contaminants. Salinity directly impairs root growth, producing a dysbiotic rhizosphere that favors a distinct microbial consortium (e.g., *Thermobifida* and xenobiotic-metabolizing taxa). Concurrent accumulation of residual pharmaceuticals and pesticides is associated with enrichment of microbial pathways related to “Human Diseases,” notably drug resistance, suggesting the microbiome both responds to and may amplify contaminant risks. This creates a stress-contaminant feedback loop in which abiotic stress weakens plant health, alters rhizodeposition, increases contaminant bioavailability, and further shifts microbiome toward a risk-indicative state.

From rhizosphere dynamics, we propose a biologically informed framework to optimize compost use. First, stress-responsive microbial taxa (e.g., *Flavisolibacter, Lysobacter*) and defense-related plant metabolites (e.g., ingenol) can serve as early-warning bioindicators to define site-specific application thresholds. Second, stimulation of pollutant-degrading genera (*Novosphingobium, Mycolicibacterium*) together with plant detoxification pathways indicates that moderate compost application can enhance the rhizosphere’s intrinsic remediation capacity. Finally, the persistent negative impact of early root damage underscores that application rates should be optimized to preserve seedling-stage root vitality and long-term rhizosphere integrity rather than to maximize transient aboveground biomass.

Admittedly, this study has certain limitations. We employed a single sludge-compost source and one depleted soil type under controlled conditions. Compost composition, soil properties, climate, and management practices may alter responses and threshold values. Consequently, the dose threshold reported here is context-specific and requires validation across multiple compost types, soil backgrounds, and longer-term field trials.

## 5 Conclusion

This study shows that sludge compost is a dose-dependent amendment: its agronomic benefits and environmental risk are determined by application rate. Moderate application (≤15% w/w) establishes a virtuous cycle, improving soil fertility, promoting a beneficial plant-associated microbiome, and enhancing stress resilience via coordinated metabolomic responses. Excessive application induces a vicious cycle of salinity stress, root impairment, and potential contaminant accumulation, accompanied by a shift toward a risk-indicative microbial community. Optimizing sludge compost rates is therefore essential for circular agriculture: restoring degraded soils through beneficial plant-microbe interactions while minimizing contaminant risks. Future work should prioritize long-term field validation of thresholds and mechanisms and the development of pretreatment methods to lower contaminant loads. Incorporation rhizosphere bioindicators into management frameworks will enable site-specific, sustainable compost use.

## Data Availability

The datasets presented in this study can be found in online repositories. The names of the repository/repositories and accession number(s) can be found in the article/Supplementary material.

## Footnotes

1
www.illumina.com
