# Supplementary material for: Sludge compost: a double-edged sword for depleted soil restoration revealed by integrated multi-omics analysis
Source: Front Microbiol. 2026 May 22;17:1731456. doi: 10.3389/fmicb.2026.1731456 (PMC13277405; doi:10.3389/fmicb.2026.1731456)
Supplement: Supplementary file 1 [file Table_1.DOCX]

Supplementary information

# 1 Supplementary Table

This supplementary information contains 9 tables (Table S1, S2, S3, S4, S5, S6, S7, S8, S9).

Table S1 Characteristics of composts and soils

|  | WW | pH | EC | SOM | TN | TP | TK | AN |
| --- | --- | --- | --- | --- | --- | --- | --- | --- |
|  | % |  | mS/cm | % | % | % | % | g/kg |
| Composts | 46.5±0.2 | 7.21 | 2.93±0.05 | 55.3±2.5 | 2.41±0.35 | 0.48±0.09 | 0.87±0.05 | 3.12±0.12 |
| Soils | 15.4±0.5 | 6.92 | 0.13±0.02 | 1.3±0.1 | 0.033±0.003 | 0.012±0.002 | 1.69±0.02 | 0.048±0.007 |

Abbreviation: wet weight (WW), electrical conductivity (EC), soil organic matter (SOM), total nitrogen (TN), available nitrogen (AN), total phosphorus (TP), available phosphorus (AP).

Table S2 Response of soil quality to varying rates of compost application at heading stage. **A)** TK contents in soil; **B)** AK contents in soil.

| **A)** | TK contents in soil (g/kg) | | |  | **B)** | AK contents in soil (g/kg) | | |
| --- | --- | --- | --- | --- | --- | --- | --- | --- |
|  | CF | CF+C | C |  |  | CF | CF+C | C |
| S1 | 16.9±0.5 | 16.2±0.7 | 16.2±0.5 |  | S1 | 0.006±0.000a | 0.033±0.005b | 0.034±0.005b |
| S3 | 16.9±0.4 | 17.9±0.5 | 16.1±0.2 |  | S3 | 0.008±0.002a | 0.038±0.004b | 0.031±0.007b |
| S5 | 17.5±0.3 | 17.0±0.6 | 15.9±0.1 |  | S5 | 0.006±0.001a | 0.035±0.003b | 0.031±0.001b |
| S7 | 16.7±0.2 | 15.4±0.3 | 15.8±0.6 |  | S7 | 0.004±0.001a | 0.032±0.006b | 0.026±0.004b |

Table S3 Environmental parameters in seedling phase

| Sample  ID | TP  g/kg | AP  g/kg | TN  g/kg | AN  g/kg | EC  mS/cm |
| --- | --- | --- | --- | --- | --- |
| C6 | 4.82 | 0.724 | 24.10 | 3.12 | 2.93 |
| S1 | 0.30 | 0.010 | 0.36 | 0.05 | 0.13 |
| S2 | 0.58 | 0.026 | 1.34 | 0.17 | 0.33 |
| S3 | 0.78 | 0.069 | 2.20 | 0.28 | 0.50 |
| S4 | 1.02 | 0.072 | 3.14 | 0.41 | 0.77 |
| S5 | 1.28 | 0.074 | 3.39 | 0.45 | 1.05 |
| S6 | 1.55 | 0.068 | 4.10 | 0.52 | 1.19 |

Table S4 VIF value of the selected parameters in seedling phase

|  | AP | EC |
| --- | --- | --- |
| VIF value | 9.04161 | 9.04161 |

Table S5 Environmental parameters in heading phase

| Sample  ID | Root | TN  g/kg | TP  g/kg | TK  g/kg | AN  g/kg | AP  g/kg | AK  g/kg |
| --- | --- | --- | --- | --- | --- | --- | --- |
| H2 | 0% | 2.43 | 0.707 | 16.20 | 0.285 | 0.033 | 0.173 |
| H3 | 0% | 2.14 | 0.646 | 16.20 | 0.204 | 0.034 | 0.172 |
| H4 | 10% | 0.58 | 0.276 | 16.90 | 0.046 | 0.008 | 0.085 |
| H5 | 10% | 2.90 | 0.753 | 17.90 | 0.334 | 0.038 | 0.171 |
| H6 | 10% | 1.95 | 0.655 | 16.10 | 0.251 | 0.031 | 0.178 |
| H7 | 20% | 1.75 | 0.274 | 17.50 | 0.038 | 0.006 | 0.087 |
| H8 | 20% | 2.77 | 0.803 | 17.00 | 0.331 | 0.035 | 0.183 |
| H9 | 20% | 1.72 | 0.724 | 15.90 | 0.179 | 0.031 | 0.166 |
| H10 | 30% | 1.67 | 0.298 | 16.70 | 0.039 | 0.004 | 0.076 |
| H11 | 30% | 2.48 | 0.769 | 15.40 | 0.230 | 0.032 | 0.167 |
| H12 | 30% | 1.63 | 0.709 | 15.80 | 0.142 | 0.026 | 0.149 |

Table S6 VIF value of the selected parameters in heading phase

|  | Root | TN | TP | TK |
| --- | --- | --- | --- | --- |
| VIF value | 1.09681 | 3.30096 | 3.91648 | 1.94936 |

Table S7 162 differential metabolites of Multiple groups (VIP>2.0)

| Category | Metabolites |
| --- | --- |
| Plant secondary metabolites  (48) | 16-Hydroxyhexadecanoic acid, Humulinic acid A, SECURININE, Rutarin, Cajaisoflavone, Xanthone, Cucurbitacin D, Nuatigenin, Leucodopachrome, Xi-Linalool 3-[rhamnosyl-(1->6)-glucoside], Octadecyl ferulate, 3-beta-Hydroxy-4-beta-methyl-5-alpha-cholest-7-ene-4-alpha-carboxylate, (+)-Dehydrovomifoliol, 3-trans-p-Coumaroylrotundic acid, Albafuran A, Echinocystic acid, Phaseol, Harpagoside, Cynaroside A, Physalin D, Wyeronic acid, Ingenol, Thial-1-Propene-1-thiol S-oxide, 8-Oxoguanosine, Cenisertib, Isopimpinellin, Monocrotaline, Isobergapten, 2-Hydroxyarctiin, S-Japonin, 13-Hydroxy-7,14-Labdadien-6-one, 3,4',7-Trihydroxyflavone, Genipin, Cis-Melilotoside, Secoeremopetasitolide A, Melilotoside, Indolepyruvate, Gynocardin, Pyrrhoxanthinol, Cyclomorusin, Toxin FS2, Gitoxin, Acoric acid, Santamarin, 1-Hexanol arabinosylglucoside, (-)-Salsoline, 2',3,4',5-Tetrahydroxy-4-prenylstilbene, (10betaH,11xi)-11-Hydroxy-13-nor-6-eremophilen-8-one |
| Pharmaceuticals and pesticides  (40) | 3-(4-(4-Chlorophenyl-4-hydroxypiperidino)methyl)indole, MG(PGF2alpha/0:0/0:0), 4-Heptyloxyphenol, Irinotecan, N-((2-(2-Fluoroethoxy)phenyl)methyl)-N-(4-phenoxy-3-pyridinyl)acetamide, N,N'-Di-1,2,3,4-Tetrahydroacridin-9-Ylheptane-1,7-Diamine, 2,8-Dibenzylcyclooctanone, Pindone, Laninamivir, Niflumic Acid, Cappariloside A, Pyridin-4-ylmethyldiazene, Thiamethoxam, Clothianidin, (3-Methoxy-4-phosphonooxyphenyl)-oxomethanesulfonic acid  Paliperidone, Cinobufotalin, Prostratin, Pravastatin lactone, Acetildenafil, Cndac, Allopregnanolone, Valsartan Acid, Misoprostol acid, 3'-Hydroxyropivacaine, Ecgonine, Quinidine, Felbamate, 3,5-dichlorosalicylic acid, 4-Oxofenretinide, Valganciclovir, (S)-, Filgotinib, 2-Fluoroadenosine, N(2)-phenylacetyl-L-glutaminate, Betaxolol, Quinapril, Etiracetam, 10-Hydroxycarbazepine, Carboprost, Pelargonic acid |
| Sugar alcohols and amino acids  (34) | Carrageenan, potassium salt of, 1-Acetoxy-2-hydroxy-16-heptadecyn-4-one, Butyryl-L-carnitine, Urea glutamate, N-alpha-Benzoyl-L-arginine, 4-Methyl-5-Thiazoleethanol, Isopropyl beta-D-glucoside, Alpha-D-Xylopyranosyl-(1->6)-beta-D-glucopyranosyl-(1->4)-D-glucose, 8-[(2R,3S)-3-(8-Hydroxyoctyl)oxiran-2-yl]octanoylcarnitine, 2,3-dimethylidenepentanedioylcarnitine, Aspartame, 3-Cyanoalanine, Val Trp, Valylhydroxyproline, Isoleucylhydroxyproline, Hydroxyprolyl-Tyrosine, Glutaminylisoleucine, Tyr-Leu-Tyr-Glu-Ile-Ala-Arg, Ac-Tyr-OEt, L-Hypoglycin A, Phenylalanylasparagine, 1-Piperideine-2-carboxylic acid, Ile Gly Leu, Asp Pro Ile, Ala Pro Phe, Methionyl-Alanine, Phe Ala Ala, Tryptophyl-Gamma-glutamate, Ile Phe, Prolyl-Glutamine, Phe Ile, Glutamic acid glutamate, Glycylleucine, 4-Methylthio-2-Oxobutanoic Acid |
| Organic acids and lipids  (18) | 14-Hydroperoxy-H4-neuroprostane, 8-Hydroxy-5,6-octadienoic acid, DG(PGJ2/13:0/0:0), 10-(2,3-Dihydroxypropoxy)-10-oxodecanoic acid, Eicosopentanoic acid, SM(d17:1/PGJ2), PI(16:0/22:2(13Z,16Z)), N-Acetylgalactosamine 4-sulphate, LysoPC(18:1(11Z)/0:0), PC(24:0/22:6(4Z,7Z,10Z,12E,16Z,19Z)-OH(14)), PE(TXB2/18:1(9Z)), PG(17:1(9Z)/0:0), Deoxycholic Acid, PE(22:6(5Z,8E,10Z,13Z,15E,19Z)-2OH(7S, 17S)/P-18:1(9Z)), PE(PGJ2/DiMe(11,5)), 4-Oxododecanedioic Acid, Undecanedioic acid, 2-(1-Ethoxyethoxy)propanoic acid |
| Microbial secondary metabolites (7) | Milbemycin A4, Milbemycin alpha6, Aminopentol, Nigerloxin, Spongothymidine, Filipin II, Citreoviridin |
| Vitamins  (5) | Ercalcitriol, Folic acid, Secalciferol, 6-Hydroxymethylpterin, Pyridoxamine 5'-phosphate |
| Others  (10) | 4-Hydroxy-3-methyl-2-(2-propynyl)-2-cyclopentene-1-one, Bolton-Hunter reagent, Heptyl 4-hydroxybenzoate, Involucrin, Q-100035, Oxidized dithiothreitol, 11-Maleimidoundecanoic acid, Sudan II, (4S,5S)-1,2-Dithiane-4,5-diol, 6-Ketoestriol |

Table S8 48 kinds of plant secondary metabolites (VIP>2.0)

|  | Metabolites |
| --- | --- |
| Terpenoids (14) | S-Japonin, Cucurbitacin D, (+)-Dehydrovomifoliol, Secoeremopetasitolide A, 3-trans-p-Coumaroylrotundic acid, 13-Hydroxy-7,14-Labdadien-6-one, Genipin, Pyrrhoxanthinol, Santamarin, Ingenol, Echinocystic acid, Acoric acid (10betaH,11xi)-11-Hydroxy-13-nor-6-eremophilen-8-one, Toxin FS2 |
| Flavonoids (5) | Rutarin, Cajaisoflavone, Cynaroside A, 3,4',7-Trihydroxyflavone, Cyclomorusin |
| Phenylpropanoids (4) | Isopimpinellin, Isobergapten, Phaseol, Melilotoside |
| Glycosides (9) | Nuatigenin, 2-Hydroxyarctiin, Xi-Linalool 3-[rhamnosyl-(1->6)-glucoside], 8-Oxoguanosine, Cis-Melilotoside, Gynocardin, Gitoxin, Harpagoside, 1-Hexanol arabinosylglucoside |
| Alkaloids (5) | SECURININE, Monocrotaline, Leucodopachrome, Indolepyruvate, (-)-Salsoline |
| Others (11) | 16-Hydroxyhexadecanoic acid, Humulinic acid A, Cenisertib, Xanthone, 3-beta-Hydroxy-4-beta-methyl-5-alpha-cholest-7-ene-4-alpha-carboxylate, Physalin D, Wyeronic acid, Thial-1-Propene-1-thiol S-oxide, Octadecyl ferulate, Albafuran A, 2',3,4',5-Tetrahydroxy-4-prenylstilbene |

Table S9 40 kinds of pharmaceuticals and pesticides (VIP>2.0)

|  | Metabolites |
| --- | --- |
| Pharmaceuticals (34) | Misoprostol acid, 3'-Hydroxyropivacaine, Quinidine, Felbamate, Quinapril, 4-Oxofenretinide, Valganciclovir, (S)-, Filgotinib, 2-Fluoroadenosine, Betaxolol, Valsartan Acid, Allopregnanolone, 4-Heptyloxyphenol, N(2)-phenylacetyl-L-glutaminate, Cinobufotalin, Acetildenafil, Etiracetam, Cappariloside A, Pravastatin lactone, Niflumic Acid, Cndac, 3-(4-(4-Chlorophenyl-4-hydroxypiperidino)methyl)indole, N,N'-Di-1,2,3,4-Tetrahydroacridin-9-Ylheptane-1,7-Diamine, Laninamivir, MG(PGF2alpha/0:0/0:0), Pyridin-4-ylmethyldiazene, Paliperidone, Irinotecan, Carboprost, 2,8-Dibenzylcyclooctanone, 10-Hydroxycarbazepine, 3,5-dichlorosalicylic acid, Prostratin, Ecgonine |
| Pesticides (6) | N-((2-(2-Fluoroethoxy)phenyl)methyl)-N-(4-phenoxy-3-pyridinyl)acetamide, (3-Methoxy-4-phosphonooxyphenyl)-oxomethanesulfonic acid, Pindone, Thiamethoxam, Clothianidin, Pelargonic acid |

# 2 Supplementary Figure

This supplementary information contains 10 figures (Fig. S1, S2, S3, S4, S5, S6, S7, S8, S9, S10).

(A)

(B)


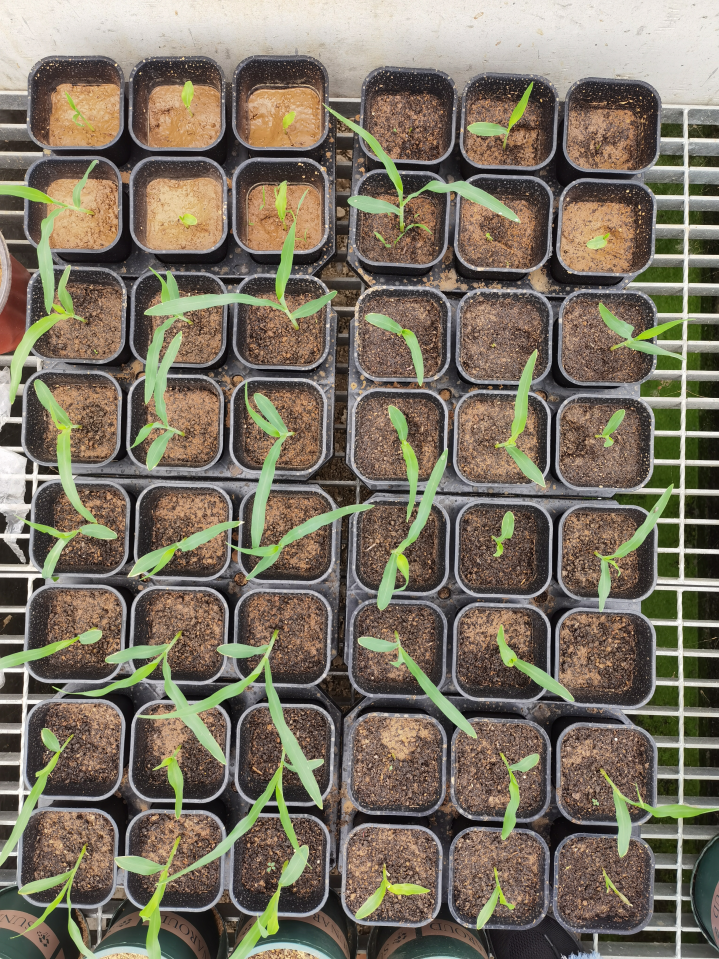

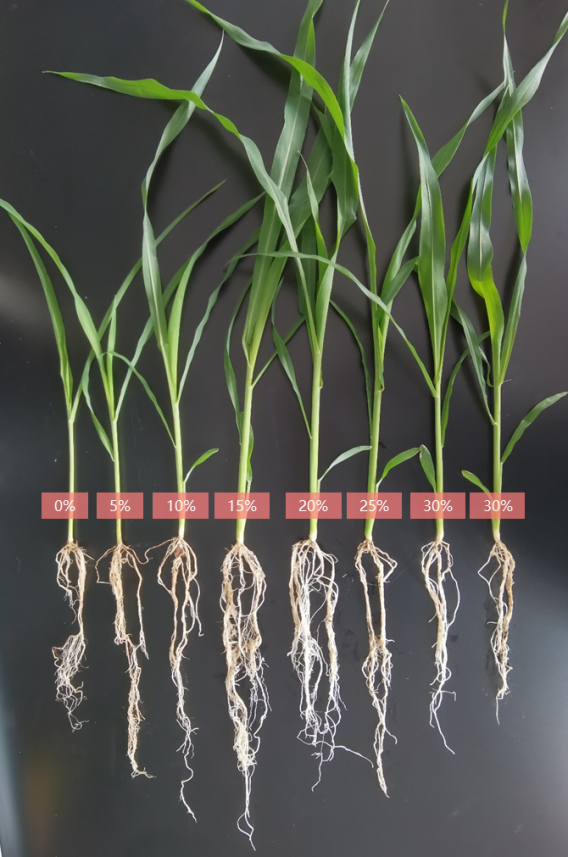


Figure S1 Maize grown at seedling phase **A)** comprising eight treatment groups with compost amendment varying from 0 to 35% (w/w), each with six replicates (n=6); **B)** root development situation of each group after 30 days’ growth.


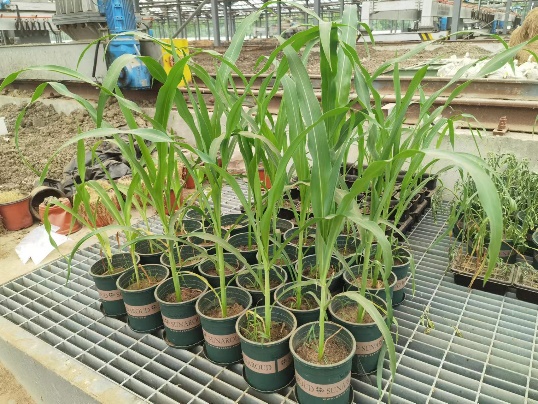


Figure S2 Maize grown at heading phase consisting of three treatment groups: **1)** chemical fertilizer only (CF); **2)** a combination of chemical fertilizer and composted material (CF+C) and **3)** composted material (C)

Figure S3 Response of maize growth to varying rates of compost application at seedling stage. **a)** stem diameter; **b)** Chlorophyll.

Figure S4 Microbial abundance and diversity index analysis for **a)** Bacteria and **b)** Fungi

Figure S5 Analysis of taxonomic composition at the **a)** domain and **b)** phylum level

Figure S6 Differential metabolite abundance profiles across treatment groups. **a)** Terpenoids; **b)** Flavonoids; **c)** Phenylpropanoids; **d)** Glycosides; **e)** Alkaloids; **f)** Others.

Figure S7 Correlation analysis of microbial taxa and plant secondary metabolite abundance.

(b)

(a)

(c)


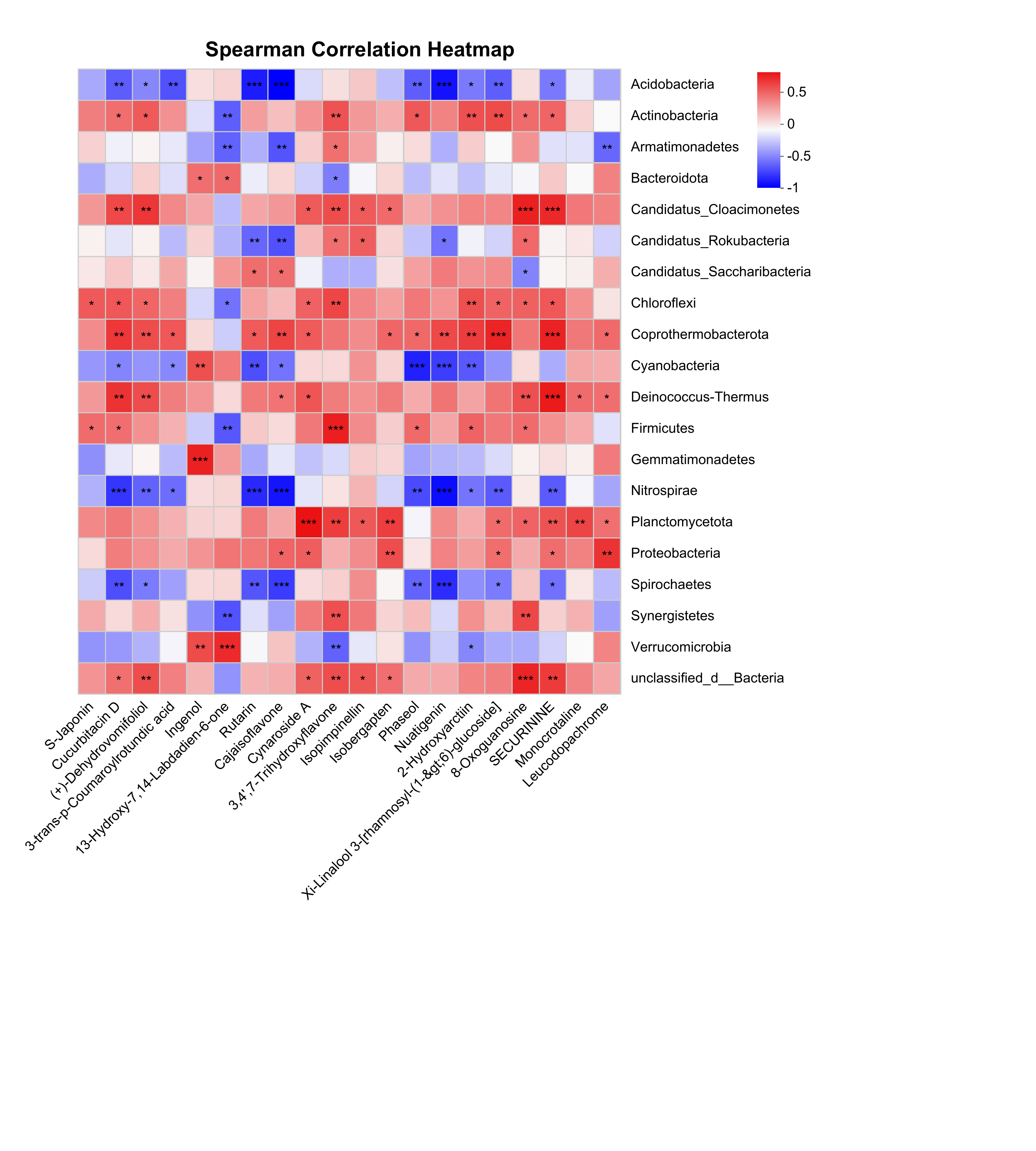

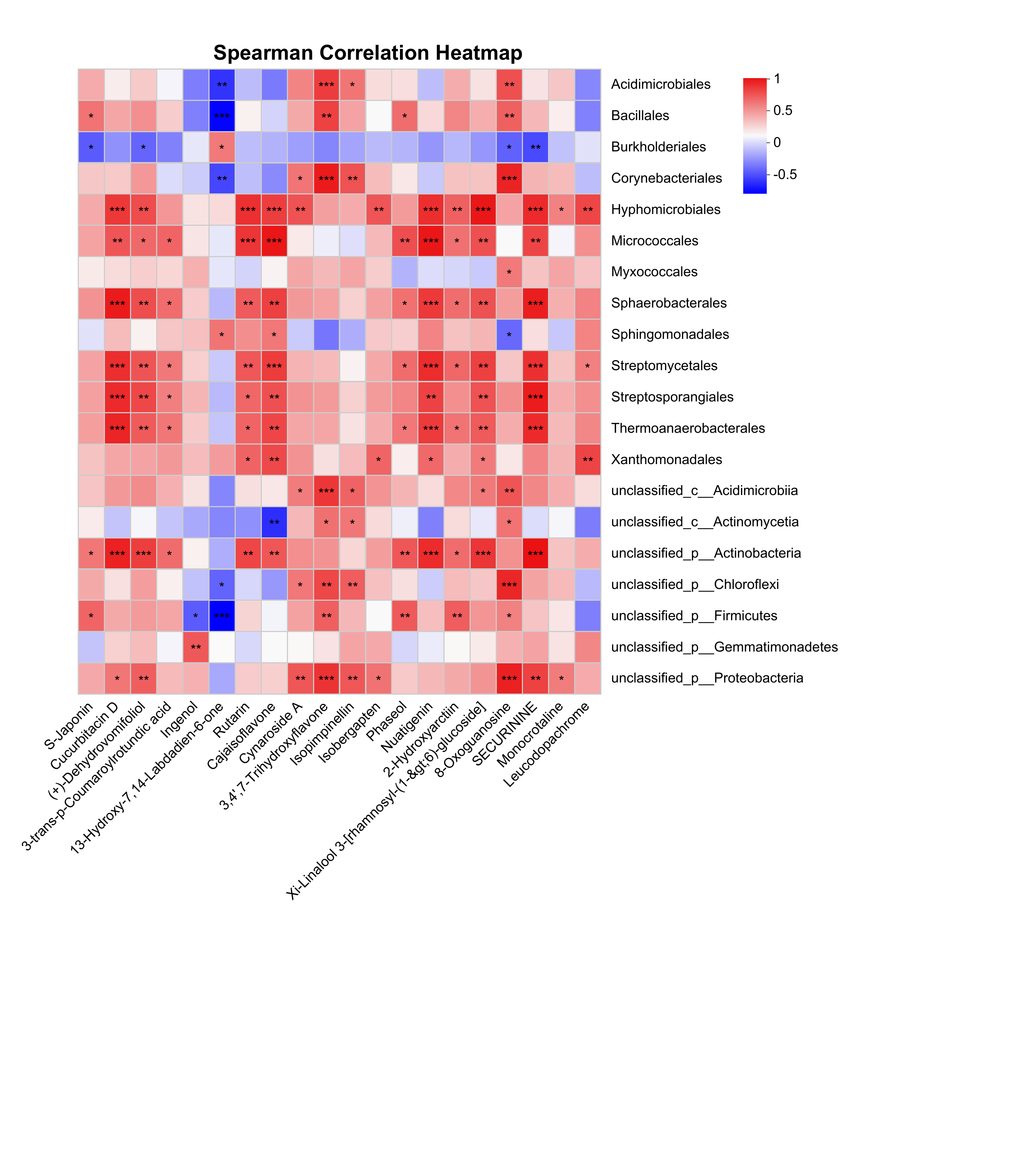

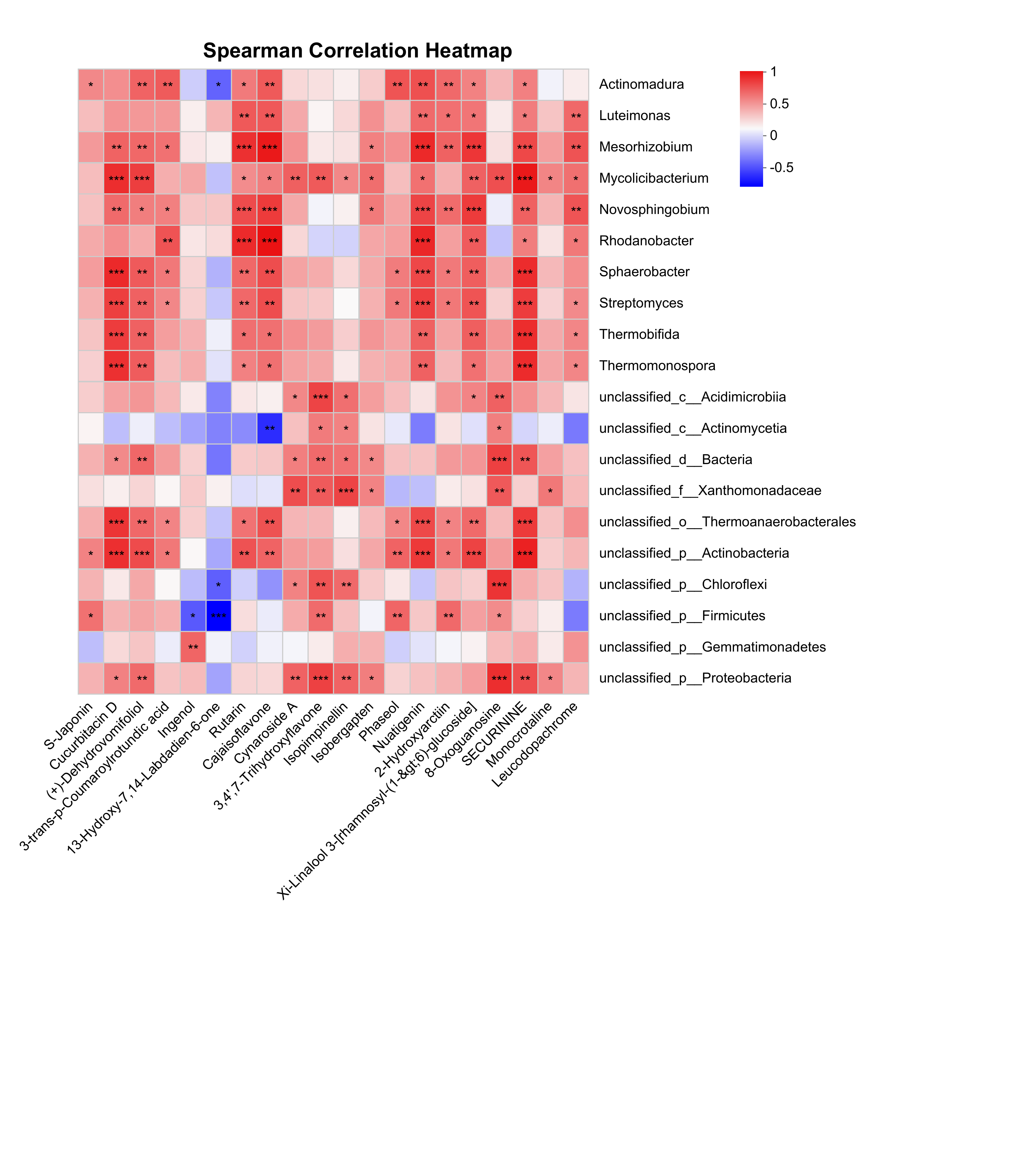


Figure S8 Analysis on spearman correlation heatmap on **a)** phylum level; **b)** order level

and **c)** genus level of Bacteria

(c)

(b)

(a)


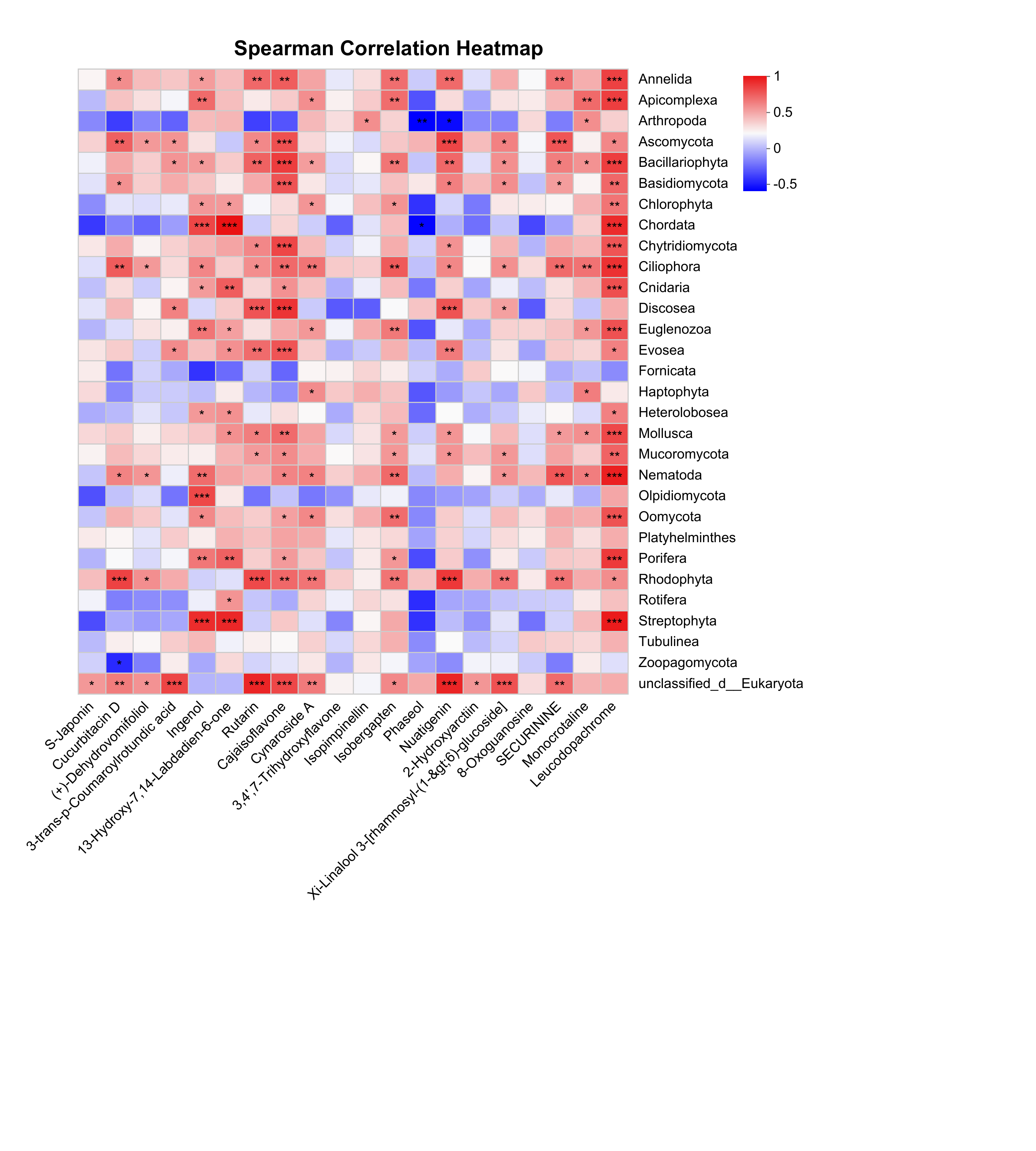

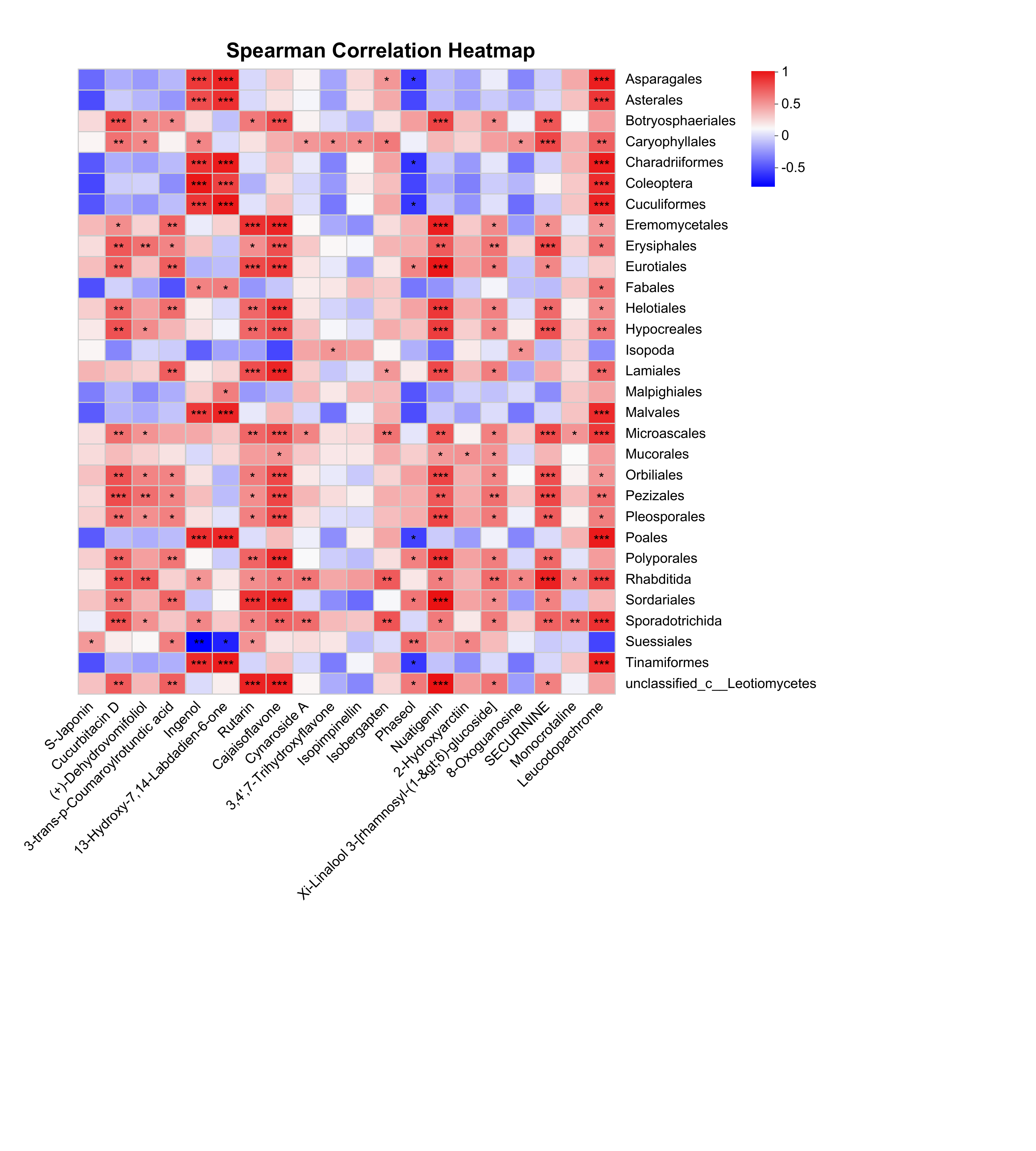

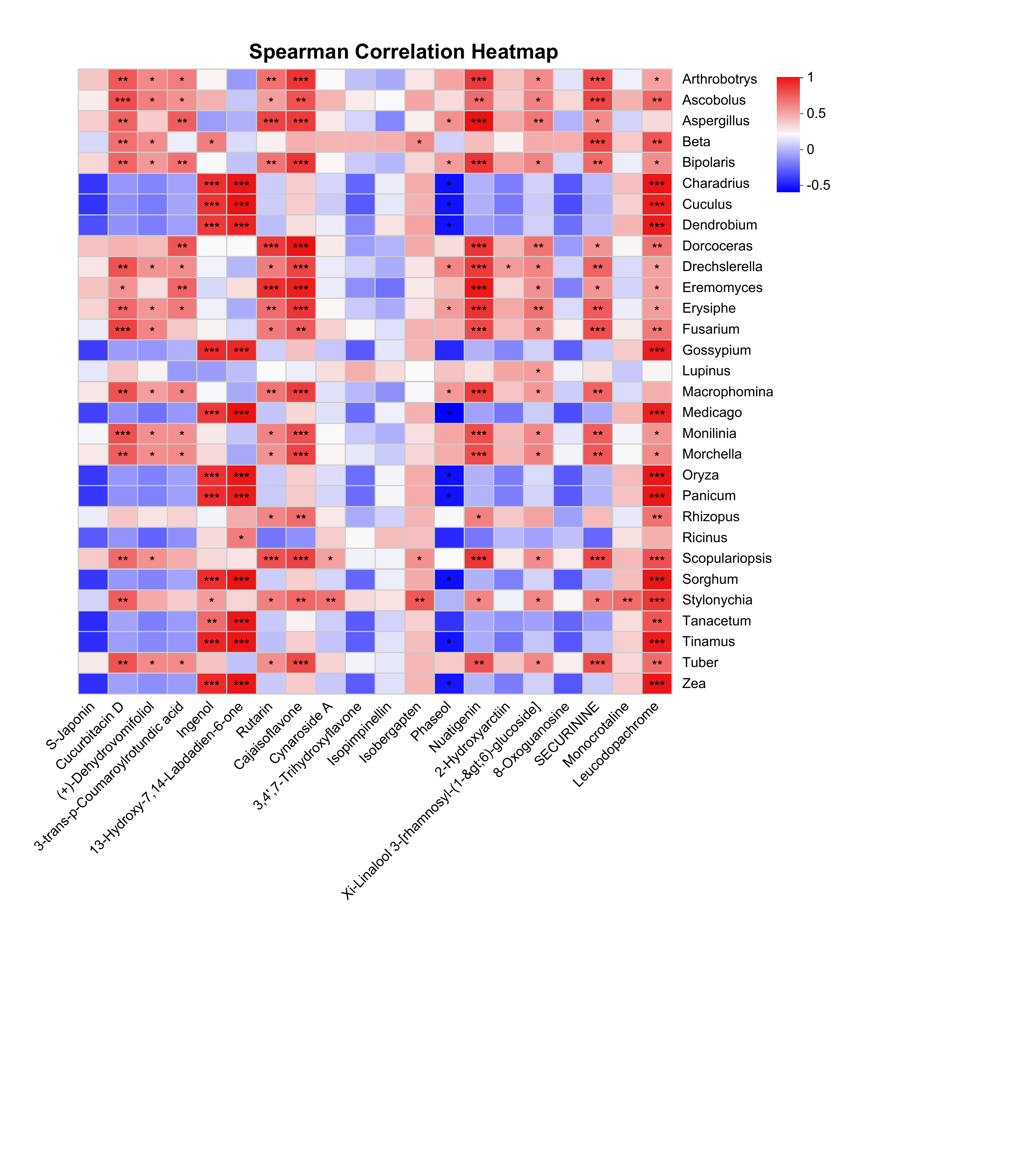


Figure S9 Analysis on spearman correlation heatmap on **a)** phylum level; **b****)** order level

and **c)** genus level of Fungi

Figure S10 KEGG pathway analysis of differential metabolites
